# Assessing the Effect of First-time Police Contact on Internalizing Problems Among Youth in Zurich, Switzerland: A Quasi-experimental Analysis

**DOI:** 10.1007/s10964-024-01986-9

**Published:** 2024-04-25

**Authors:** Amy Nivette, Laura Bechtiger, Denis Ribeaud, Lilly Shanahan, Manuel Eisner

**Affiliations:** 1 Department of Sociology, Utrecht University, Utrecht, The Netherlands; 2 Jacobs Center for Productive Youth Development, University of Zurich, Zurich, Switzerland; 3 Department of Psychology, University of Zurich, Zurich, Switzerland; 4 Institute of Criminology, University of Cambridge, Cambridge, UK

**Keywords:** Police contact, Internalizing problems, Mental health, Quasi-experimental design

## Abstract

Growing evidence suggests that experiences with police are associated with a range of negative mental health problems among youth. This study examined the impact of negative police contact on changes in adolescent internalizing problems, measured by anxiety and depression. Six waves of data from a longitudinal study in Zurich, Switzerland were used in order to assess the direct relations between first reported police contact in the years prior to the survey moment and internalizing problems at the time of the survey and follow-up waves. The sample consists of a cohort of youth (max *n* = 1353, 49.4% females) spanning ages 11 to 24 (mean age and SD at each wave = 11.32 (0.37), 13.67 (0.36), 15.44 (0.36), 17.45 (0.37), 20.58 (0.38), 24.46 (0.38)). Specifically, difference-in-differences techniques for multiple time periods were employed to assess the average treatment effects for the treated population (first contact with police) compared to those who were never treated (never had contact). Across all models, police contact did not lead to an increase in internalizing problems. These results diverge from previous studies mostly conducted in the United States, and possible explanations including differences in historical contexts of policing, juvenile justice, health care, and dosage of intrusive contacts are discussed.

## Introduction

Growing evidence suggests that experiences with police are associated with a range of negative mental health and behavioral outcomes among youth, including elevated psychological distress (Del Toro et al., 2019), anxiety (Geller et al., 2014), depression (Hirschtick et al., 2020), post-traumatic stress disorder (Jackson et al., 2019), sleep disorders (Jackson & Testa, 2022), and poorer self-rated health outcomes (Jahn et al., 2021). Negative encounters with the police are also associated with negative affective responses, such as feelings of anger, distress, and fear (McLeod et al., 2020). This stress can persist long after the encounter, adversely influencing psychological well-being and health (Testa et al., 2021). These effects of police contact could be particularly relevant during adolescence, a period of substantial social, psychological, and biological changes (Granot & Tyler, 2019) during which many mental health problems (Patton et al., 2016), and also stressor exposure first emerge (McLaughlin & Hatzenbuehler, 2009). To date, most studies on the effects of police contact tend to assess between-individual differences in mental health (e.g., cf. Del Toro et al., 2022). One major concern in these studies is that temporal ordering and baseline measures of mental health are not always accounted for in the design, increasing the risk of bias. In addition, the majority of studies on the adverse effects of police contact on youth mental health have been conducted in the United States, where policing is often characterized as aggressive, frightening, and discriminatory towards ethnic and other minority groups (Braga et al., 2019), and possibly life-threatening. More research in different policing and social institutional contexts is needed to understand when and why police contact has negative effects on mental health outcomes. The current study uses within-person longitudinal data in a context with youth laws and policing practices that are designed for de-escalation and education to examine the impact of negative police contact on changes in adolescent internalizing problems (i.e., anxiety and depression). In order to do so, data were drawn from an ongoing longitudinal study of an ethnically diverse sample of youth from Zurich, Switzerland - the Zurich Project on Social Development from Childhood to Adulthood (z-proso).

### Literature Review

The prevalence of internalizing problems such as feelings of depression and anxiety increases at the transition to adolescence (Costello et al., 2011). The processes involved in this increase are multifaceted, spanning from biological influence factors related to pubertal development (e.g., hormonal changes and accompanying neurodevelopment), to emotional and cognitive maturation processes, to environmental influences (Hankin, 2015). In particular, understanding which (preventable) exposure to adverse experiences and stressful life events are related to increased internalizing problems is relevant (March-Llanes et al., 2017). Within this broad body of research, one adverse experience that has received comparatively less attention so far in the literature on emerging internalizing problems in adolescence is contact with the police.

Police contact could induce internalizing problems, such as anxiety and depression, for a number of reasons. First, police contact may be a result of externalizing problems, or in this case, criminal behavior, which is independently associated with peer rejection, victimization, and lower self-esteem. These phenomena, in turn, can manifest in feelings of anxiety or depression (Moilanen et al., 2010). This is inherently a within-individual process, as changes in externalizing behavior leading to police contact are expected to be associated with changes in internalizing symptoms. Second, the interaction with the police can be potentially traumatic (Gearhart et al., 2022), which can lead to physiological and emotional stress responses, including anger, shock, and sadness (McLeod et al., 2020). Especially in the US, contact with police could induce fear for one’s life, which can increase the risk of developing post-traumatic stress disorder (PTSD), anxiety, and depression. In addition, the adverse life events literature shows that major life events, or non-normative stressors, tend to be associated with increased risk of internalizing problems (Kim et al., 2003), in particular depression and anxiety (McLaughlin & Hatzenbuehler, 2009). Stress from adverse life events can lead to emotional dysregulation, or reduce the ability to monitor, evaluate, and modify emotional reactions to achieve goals (Thompson, 1994), which increases vulnerability to the effects of stress (McLaughlin & Hatzenbuehler, 2009).

There is relatively consistent evidence to suggest that (direct or vicarious) police contacts characterized by intrusiveness, use of threats, and racist language are associated with greater emotional distress during the stop (Jackson et al., 2019) and reported anxiety and depression after experiencing or witnessing the stop (Hirschtick et al., 2020). The context in which individuals were stopped or contacted by the police varied across studies. For example, the Fragile Families and Child Wellbeing Study measures whether the youth at age 15 was ever stopped by the police, when and where, followed by retrospective measures of treatment during the stop (McFarland et al., 2019). Others have used the lifetime number of police stops (e.g., Hirschtick et al., 2020), frequency of being stopped by the police in the past 6 months (Del Toro et al., 2019), police victimization (McLeod et al., 2020), and the prevalence of being warned, cautioned or arrested by police (Jackson et al., 2021). While the measures of police contact vary, most can be similarly described as “negative” contact, wherein the interaction is typically police-initiated and the treatment is unfair, intrusive or results in negative consequences such as arrest. For example, treatment such as handcuffing (without arrest), frisking, using harsh language, racial slurs, and the threat or use of force were associated with distress during the stop (Jackson et al., 2019). By contrast, one study found that more “positive” treatment, defined by procedural justice, was associated with lower anxiety and PTSD among a sample of New York City residents, and that procedurally just treatment may to some extent offset the negative impact of lifetime number of contacts on PTSD (Geller et al., 2014). However, based on this literature, it is not entirely clear whether and to what extent different types of contact have stronger or weaker effects on mental health outcomes. Using data from the United Kingdom, two studies examined how different types of contact (i.e., being stopped and questioned, being cautioned or warned, and being arrested) were associated with physical and mental health outcomes such as sleep (Jackson & Testa, 2022) and self-harm (Jackson et al., 2021). Their results showed that all three types of contact were associated with negative health outcomes, but that being cautioned/warned and especially being arrested had the largest impact on sleep problems and self-harm. It is therefore likely that being arrested or cautioned would generate more persistent feelings of stress compared to a single incident of being stopped on the street.

Only two studies examined within-individual associations between police contact and mental health outcomes. The first study found that police stops were associated with greater next-day psychological distress (Del Toro et al., 2022), and the second study found that being arrested was associated with more severe mental health problems reported that year (Sugie & Turney, 2017). Notably, the latter study uses a measure that closely aligns with internalizing problems, as it captures the frequency of feelings of anxiety and depression in the past month. This suggests that negative police contact is related to both between- and within-individual variation in mental health outcomes. Furthermore, the timeframes for these studies vary substantially. One study measured distress the following day (Del Toro et al., 2022), without measuring long-term changes, whereas other studies measured police contact retrospectively and assessed mental health outcomes measured at the time of the survey (e.g., Baćak & Apel, 2020). A few studies employed matching procedures to account for selection effects, but the large time gap between waves, and thus, between treatment and outcome, also means that the possibility of bias and reverse causality cannot be ruled out (Petersen et al., 2023).

### Context

Zurich is Switzerland’s largest city with ~450,000 inhabitants and ~1,500,000 inhabitants in the larger metropolitan area. Although Zurich is internationally known as a hub for the banking and finance industries, inhabitants of Zurich come from all socioeconomic backgrounds. Switzerland is an immigration country and approximately one-third of the population has a migration background. This number is even higher in larger cities, as reflected in the sample of the present study. Globally, the crime rates in Switzerland are low. Despite the low official crime rates, previous surveys suggest that delinquency among young people is common in Zurich, with 1 in 10 adolescents engaging in criminal behavior (Ribeaud & Loher, 2022) and high rates of illegal substance use in adolescence and young adulthood (Quednow et al., 2022).

The European context of policing is comparable to the US in some ways, for example, experiences of aggression and discrimination by police towards youth and ethnic minorities (Solhjell et al., 2019). However, in Western European countries there is comparatively higher trust in police and less police violence (Hirschfield, 2023). In addition, European countries such as Switzerland tend to have broader access to social “safety nets” and health care compared to the US. While these differences in context suggest that the relationship between police contact and mental health may be weaker in European contexts, some studies from the UK have nevertheless found that contact was associated with more physical and mental health problems (Jackson & Testa, 2022). In particular, one cross-national European study found that dissatisfaction with recent (in the past 2 years) police contact was associated with lower reported health, emotional ill-being, more loneliness, and unhappiness (Baćak & Apel, 2020).

The Swiss juvenile justice system is also considered a “moderate system of minimum intervention”, which places emphasis on diversion (Dünkel, 2015, pg. 9), or non-custodial punishments such as community service, education (e.g., social competence training, targeted offender programs, drug or alcohol education), fines, and judicial reprimand (Pruin et al., 2017). Trust in the police and ratings of procedural fairness are relatively comparable to other Western European countries, but higher than Eastern European countries (Staubli, 2016). Although trust in the Swiss police is overall high, research from Switzerland (Plümecke et al., 2023) and other Western (Krieg et al., 2022) and Northern European countries (Solhjell et al., 2019) suggest that youth, especially minority youth, reported feeling targeted and discriminated by the police (Dirikx et al., 2012). Negative (i.e., in relation to wrongdoing or dissatisfaction) contact was also associated with more negative perceptions of police (Nivette et al., 2020). Youth with a migration background, particularly those from Africa or the Middle East, tend to have relatively lower trust compared to non-migrant youth (Baier et al., 2019). This suggests that the context of policing, experiences of discrimination, and effects of police contact in Switzerland are comparable to other Western European countries.

## Current Study

The majority of existing research on police contact and mental health has been conducted in the United States and has focused largely on assessing between-individual differences. This study extends previous research to evaluate the association between negative police contact and within-individual changes in self-reported internalizing symptoms in a sample of urban youth in Zurich, Switzerland from ages 11 to 24 years old. Relatively few studies outside the US have examined to what extent police contact influences internalizing problems, or other mental health outcomes, over time. Based on previous research in the US and Europe, the current study hypothesizes that negative police contact in the timeframe prior to the survey will predict greater internalizing symptoms in the period following contact, adjusting for other pre-existing differences and trends. In addition, this study explores the heterogeneity of treatment over time, specifically the extent to which the treatment effect may persist over time. No specific hypotheses were made about these residual effects, but one may expect based on broader criminological literature that contacts occurring in early adolescence (i.e., ages 11–15) would have a greater and more persistent impact compared to contacts occurring in late adolescence and early adulthood (i.e., ages 17–24).

## Methods

The data for this study came from the Zurich Project on Social Development from Childhood to Adulthood (z-proso), an ongoing prospective-longitudinal study in Zurich, Switzerland. The z-proso study is based on a cohort of children who entered one of 56 primary schools in the city in 2004 when children were aged 7 years. The initial target sample of schools was selected using stratified random sampling in which disadvantaged schools were slightly over-selected. The study population is largely representative of the city of Zurich, but not of Switzerland more generally. Zurich is Switzerland’s largest city, with relatively similar crime rates compared to other major cities in the country. According to self-reported surveys of youth, the Canton of Zurich (which includes the city and surroundings) had relatively higher rates of violent and property crime in 2014 than the comparable Canton of Vaud (Ribeaud et al., 2015). All children entering first grade at sampled schools were invited to participate in the study (*N* = 1675). In the first wave, 81% of the target sample participated (*n* = 1360). The study consists of nine waves of youth interviews at ages 7, 8, 9, 11, 13, 15, 17, 20, and 24. Active parental consent was required up to age 11, between ages 13 and 15 (waves 5 and 6) active youth and passive parent consent was required, and after age 17 (waves 7+) active youth consent was required. Further information on recruitment, participation, general patterns of attrition, and materials can be found elsewhere (Eisner & Ribeaud, 2005; Eisner et al., 2019; Ribeaud et al., 2022).

The current study focuses on the most recent six waves when the majority of participants were aged 11, 13, 15, 17, 20, and 24, respectively. In wave 4 (age 11), 1,147 (69%) of the initial target sample participated. Between waves 4 and 5, attrition was 10.1% (*n* = 116), 2.6% (*n* = 36) between waves 5 and 6, 10.9% (*n* = 159) between waves 6 and 7, 14.4% (*n* = 188) between waves 7 and 8, and 9.7% (*n* = 115) between waves 8 and 9. In wave 9, 80.7% of respondents defined as “eligible” participated (*n* = 1160 out of 1437), and 75% of those who participated at wave 1 also participated in wave 9 (69.3% of the initial target sample). Notably, in waves 5 and 6, the study team was able to re-contact the entire initial target sample, and as a result, 81.6% of the target sample participated at age 13 (*n* = 1365), and 84.6% at age 15 (*n* = 1446). In relation to the key variables in the current study, an analysis of attrition showed that there was no significant relationship between first contact with police and dropping out at later waves. However, those with any police contact in waves 6, 7, and 8 were more likely to drop out in later waves (waves 8 and 9). There were no significant differences in self-reported delinquency between those who participated and dropped out. For internalizing problems, those who reported *lower* levels of internalizing problems in waves 5, 6, and 7 were more likely to drop out in later waves, but not between waves 8 and 9. Analyses using first contact may therefore be biased towards participants who have higher reported internalizing problems, while analyses using any contact may also be biased towards those who did not have any police contact during adolescence. There was no differential attrition related to participants’ engagement in delinquency.

At ages 11, 13, 15, and 17, participants completed pencil and paper questionnaires in schools or at the university outside of classroom times. At ages 20 and 24, participants completed surveys on a computer at a university research laboratory. At ages 20 and 24, participants also had the option to complete the survey online (*n* = 37 and *n* = 173, respectively). Participants were compensated for participation starting at wave 5: 30 CHF (~30 USD) at age 13, 50 CHF at age 15, 60 CHF at age 17, 75 CHF at age 20, and 150 CHF (in a lab setting) or 100 CHF (online) at age 24. Parents in wave 4 were compensated with shopping vouchers worth 50 CHF.

### Analytical Approach

The goal of this study was to assess the impact of police contact on internalizing symptoms, with attention to potential causal effects. Typically, fixed effects estimators may be used to assess contemporaneous effects while accounting for unobserved person- and time-specific heterogeneity (i.e., one- or two-way fixed effects) (Imai & Kim, 2021). However, these approaches can be biased when effects are heterogeneous across individuals and time, when comparing newly treated to already treated individuals, and when there may be feedback effects from the outcome of the treatment (Nguyen, 2020). As such, this study implements recently developed analytical techniques that allow one to estimate average treatment effects for the treated population [ATT]. Namely, recent extensions of difference-in-differences [DiD] for multiple time periods were used, which allows for causal inference in the presence of treatment effect heterogeneity and dynamic effects, and can incorporate covariates in the estimation process (Callaway & Sant’Anna, 2021b). This approach compares the change in internalizing problems experienced by the treated group (i.e., first reported police contact) to the change experienced by the control group (i.e., those never reporting police contact). Put simply, the ‘canonical’ DiD estimates the difference in changes in internalizing problems before and after an individual’s first contact with the police (treatment) compared to the changes among those who have never been in contact with the police (control) (Cunningham, 2021). This approach also allows one to account for variation in treatment over time, as some respondents would have experienced their first contact with police at different points during adolescence. Specifically, the group-time average treatment effect is a disaggregated estimate of the ATT for a particular group *g* (a “group” reflects the time period of first treatment) at a particular time period *t* (wave), denoted as ATT(*g,t*). This means that it is possible to evaluate heterogeneity in treatment effects over time following the first police contact, as well as provide an overall treatment effect (Callaway & Sant’Anna, 2022).

One important assumption within DiD approaches is the parallel trend assumption, which specifies that there should be no difference between the treated and untreated group if no treatment had occurred (Huntington-Klein, 2022). This assumption cannot be directly tested since the counterfactual is unknown (i.e., trends among treated if untreated), but one can conduct “pre-tests”, or pre-treatment comparisons, which provide some indication as to whether trends already deviate significantly prior to treatment (Callaway & Sant’Anna, 2022). Recent extensions have also shown that it may be sufficient to use the conditional parallel trend assumption (Callaway & Sant’Anna, 2021b), which assumes that respondents with the same characteristics would follow the same trend in internalizing problems in the absence of police contact (treatment) (Roth et al., 2023). Applying the doubly robust procedure, both a generalized propensity score using theoretically relevant time-varying and invariant covariates and outcome regressions were estimated (Sant’Anna & Zhao, 2020). The generalized propensity score therefore represents the probability of being first treated conditional on covariates, or in the never-treated group at a particular wave (Callaway & Sant’Anna, 2021b). For time-varying covariates, the matching procedure uses values measured during the “base period”, which refers to the period immediately prior to treatment or prior to the current period for the pre-treatment time frame (Callaway & Sant’Anna, 2022).

The “untreated” group in this model consisted of respondents who, according to the current measures, have not reported negative contact with the police in any of the six waves. In order to have a baseline measure of untreated respondents in the first wave, participants who reported having any prior police contact at age 11 (*n* = 30) were excluded. In addition, participants who were treated (i.e., had first police contact) in the most recent wave (9, age 24) were excluded for two reasons: first, the number of those treated was relatively small (*n* = 28), which can lead to unstable estimates (Callaway & Sant’Anna, 2022), and second, the aim was to estimate both short-term and long-term effects which was not possible among those treated in wave 9.

Two types of average treatment effects of police contact on internalizing problems were estimated. First, the group-time ATT was estimated, which provides estimates of the treatment effect for each group (i.e., first contact at different age periods) for all post-treatment periods. Second, an overall ATT using a dynamic approach and a group approach was estimated. The dynamic approach provides average treatment effects over different lengths of exposure to treatment, whereas the group approach provides average treatment effects per group (i.e., per survey wave of first contact) (Callaway & Sant’Anna, 2021b). The group approach also provides an overall ATT that is the average of treatment effects across groups, which is recommended as a better estimate of overall ATT similar to a traditional 2×2 DiD design (Callaway & Sant’Anna, 2021a). This estimator provides a flexible option for staggered and heterogeneous treatments, which can provide less biased estimates compared to traditional two-way fixed effects DiD estimators (Baker et al., 2022).

All analyses were conducted with the “did” package (Callaway & Sant’Anna, 2021a) in R (R Core Team, 2021). This package calculates confidence intervals that are robust to multiple tests by adjusting and increasing the critical values for determining statistical significance for 95% uniform confidence intervals. Analyses were conducted using a sample of youth who have participated in at least three waves between ages 11 and 24 (*n* = 1411, 81.6% of the initial target sample), resulting in an unbalanced panel. In other words, the panel did not have an equal number of participants for each time point since some may have participated in certain waves but not others, or due to missing data. The did package accounts for an unbalanced panel by calculating the ATT(g,t) using those with complete observations in the specific time period(s) and groups (treatment and control). The N used for analyses therefore may vary across estimates for disaggregated group and time effects. Excluding those who reported police contact in waves 4 and 9, the final maximum sample for analysis was *n* = 1353.

### Measures

#### Police contact

The main “treatment” measure of police contact was a dichotomous variable that measures self-reported contact with the police (1=yes) in relation to wrongdoing in the time period prior to the survey wave (spanning 2–4 years). As such, the type of contact was assumed to occur under negative circumstances. This was measured using the wave of the first reported contact as the time of treatment (group). In order to obtain the most complete measure of those who received treatment, a number of variables available in the z-proso dataset were used: a general retrospective item in relation to wrongdoing (past 2–4 years) measured in waves 5–9, a measure of contact in relation to specific deviant behaviors (past 12 months) measured in waves 5–9, and another general retrospective item in relation to wrongdoing (past 2 years) measured in waves 4–7. More information on these three items can be found in Appendix A. If a respondent indicated they had contact using at least one measure, they were counted as “treated” at that wave. Here it is assumed that respondents could not be “un-treated” (i.e., respondents could not be un-contacted by the police) and so they were considered treated throughout the rest of the time period. The resulting sample allowed us to explore both the short- and longer-term effects of negative contact on internalizing symptoms.

Across all waves, 63.71% (*n* = 899) of participants had never been treated, i.e., they had not reported negative personal contact with the police in the time prior to any survey moment (see Table 1).^1^ As mentioned, 2.13% (*n* = 30) experienced first contact prior to wave 4 (age 11). 9.07% (*n* = 128) experienced first contact by wave 5 (age 13), 9.64% (*n* = 136) by wave 6 (age 15), 6.95% (*n* = 98) by wave 7 (age 17), 6.52% (*n* = 92) by wave 8 (age 20), and 1.98% (*n* = 28) by wave 9 (age 24). Table 1 also shows that, with the exception of wave 6 (age 15), male youth were more likely to report police contact compared to female youth. Some of the more common reasons for contact, where information is available, were in relation to fare dodging, the consumption of alcohol or tobacco underage, the consumption of cannabis, and theft.

**Table 1:** Distribution of first reported police contact (treatment) by sex and survey wave

Treatment group	Male	Female	Total (%)
Never reported police contact	403	496	899 (63.71%)
First reported police contact…			
Wave 4 (age 11)	25	5	30 (2.13%)
Wave 5 (age 13)	90	38	128 (9.07%)
Wave 6 (age 15)	68	68	136 (9.64%)
Wave 7 (age 17)	65	33	98 (6.95%)
Wave 8 (age 20)	59	33	92 (6.52%)
Wave 9 (age 24)	10	18	28 (1.98%)

#### Internalizing problems

Internalizing problems was operationalized using a combined anxiety and depression scale. Anxiety and depression were measured using an adapted version of the Social Behavioral Questionnaire (SBQ, Tremblay et al., 1991). Four anxiety and four depression items were measured across all included waves. Respondents were asked to report the frequency of a given symptom in the past month (e.g., “I felt nervous, anxious or tensed,” “I was unhappy, miserable, or distressed,” “I was sad without knowing why”) on a 5-point Likert-type scale ranging from “never” to “very often” (Murray et al., 2019). The internal reliability of the scale was good across waves (Cronbach’s α_w4_ = 0.79, α_w5_ = 0.83, α_w6_ = 0.84, α_w7_ = 0.85, α_w8_ = 0.87, α_w9_ = 0.86).

#### Covariates

Additional covariates were included that are expected to influence selection into treatment (i.e., police contact), as well as factors that may influence trajectories of internalizing problems in the absence of treatment (Abadie, 2005). Information about the selection and construction of covariates is available in Appendix A. Descriptive statistics and bivariate correlations for wave 5 can be found in Table A1.

#### Externalizing behavior

First, two measures of externalizing behavior were included, namely aggression and deviant behavior. Aggression was measured using the SBQ and is comprised of 9 items covering physical, reactive, and proactive aggressive behavior in the past 12 months. Responses were measured on a 5-point Likert-type scale ranging from “never” to “very often”, and the scale was reliable across waves (α_w4_ = 0.78, α_w5_ = 0.88, α_w6_ = 0.88; α_w7_ = 0.86; α_w8_ = 0.85; α_w9_ = 0.86). Involvement in deviant behavior was measured using a variety scale, which reflects the number of different self-reported deviant acts that the respondent engaged in. Respondents were asked whether they engaged in any of the nine deviant behaviors in the 12 months prior to the survey moment, including for example stealing from home/school/work, fare dodging, vandalism, consuming THC, shoplifting, and assault.

#### Victimization

Respondents were asked to report whether or not they were the victim of robbery, assault, or serious sexual assault. In wave 4, respondents were not asked about sexual assault victimization. In wave 9, respondents were asked about prevalence in the past 4 years, and then the number of incidents in the past 12 months. A dichotomous variable was created to reflect whether the respondent had been a victim of any violent crime at each survey time point (1 = yes).

#### Other stressful life events

In addition, an overall measure of the prevalence of other stressful life events during the study period was included. These life events were not measured at wave 4, so the variable reflects an average number of different stressful events that occurred in the time prior to wave 5 up to wave 9 (roughly ages 12–24). For each available wave, a variety score was created that captured the number of different life events, including whether or not the respondent has been hospitalized themselves, whether a parent, sibling, grandparent, or close friend has died in the time prior to the survey. The death of a sibling and grandparent in later waves was combined into a single category, and so this was applied to previous waves as well. The range of possible events is therefore 0 to 4. These scores were averaged across waves to get the average number of different stressful life events during (most of) the study period.

#### Low self-control

Low self-control consisted of 10-items adapted from Grasmick et al. (1993), which consisted of five subdimensions of two items each (i.e., impulsivity, self-centeredness, risk-seeking, preference for physical activities, and short temper). The agreement was measured on a 4-point Likert-type scale ranging from “fully untrue” to “fully true”. Reliability was acceptable across waves (Cronbach’s α_w4_ = 0.75, α_w5_ = 0.78, α_w6_ = 0.75; α_w7_ = 0.73; α_w8_ = 0.75; α_w9_ = 0.74).

#### Moral neutralization

Moral neutralization was measured using 11 items that reflect cognitive processes that work to justify deviant beliefs and behaviors within one’s moral landscape (Ribeaud & Eisner, 2015). The four mechanisms represented in the scale included cognitive restructuring (5 items), blaming the victim (3 items), distorting negative impact (2 items), and assuming the worst (1 item). Responses ranged on a 4-point Likert-type scale from “fully untrue” to “fully true” (Cronbach’s α_w4_ = 0.85, α_w5_ = 0.88, α_w6_ = 0.87; α_w7_ = 0.89; α_w8_ = 0.89; α_w9_ = 0.88).

#### Peer delinquency

Peer delinquency was measured using self-reported friends’ involvement in deviance, which was measured in all waves. Respondents could indicate whether they had up to two “best friends” and answer whether or not these friends were involved in six deviant behaviors. This included violence, theft, truancy, alcohol, smoking cigarettes, and taking drugs. In later waves, truancy referred to both skipping school and/or work, and drinking alcohol and smoking cigarettes referred to consumption on a daily basis. For each friend, a mean score was created, and if the respondent indicated two friends, the average of the two scores was taken. When respondents did not report any best friends, they were recoded as 0. As such, the scale ranged from 0 (no best friends, no deviant best friends) to 1, where any value over 0 indicates having a best friend involved in some type of deviant behavior.

#### Unstructured leisure time

Three items were used to capture the frequency of engaging in unstructured leisure activities that involve criminal behavior (i.e., doing something prohibited, shoplifting, fighting with others). Responses were measured using a 6-point scale ranging from “never” to “(almost) daily”. These items were only included in waves 4 through 8 (not in wave 9), so an overall scale was constructed reflecting the average frequency of unstructured criminal activities during the study period.

#### Socio-demographic variables

Three relatively stable covariates were included to account for socio-demographic differences between participants. Sex was coded 0 for females and 1 for males. Migrant background reflected whether the youth had at least one parent born in Switzerland (coded 0), or both parents born outside of Switzerland (coded 1). The sample is highly diverse: in waves 5 and 6 (age 13/15), about 24% of respondents had two parents who were born in Switzerland, about 27% had one parent born abroad, and 50% had both parents born abroad. The most common countries of origin were the former Yugoslavia, Germany, Portugal, Sri Lanka, Turkey, and Italy (Ribeaud et al., 2022). Socio-economic status (SES) was measured based on the primary caregiver’s occupation when the respondent was 13 or 15 years old. Codes were transformed into an International Socioeconomic Index of Occupational Status [ISEI] score (Ganzeboom et al., 1992). The respondent’s SES score was based on the highest ISEI recorded for each household. Additionally, the average level of internalizing problems across all waves was included as a relevant covariate influencing potential selection and trends.

## Results

**Fig. 1:**
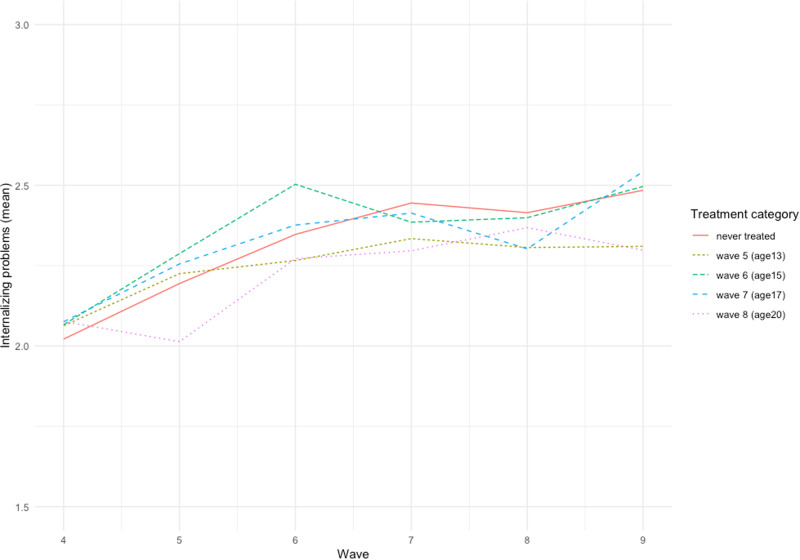
Mean levels of internalizing problems by treatment category (first reported contact with police) over time

Figure 1 shows the average levels of internalizing problems for each treatment or control group throughout the study period. Generally, the trends follow the same pattern of increasing between waves 4 (age 11) and 6 (age 15) from early to mid-adolescence, before plateauing into young adulthood. The differences between groups are relatively small, and the trends do not show any clear differences between those treated compared to never treated participants.

Before the DiD analyses, independent samples t-tests were conducted to estimate the difference in mean internalizing problems for each treatment wave (waves 5–8). For the most part, there were no significant differences between treated (i.e., those who report first police contact in that wave) and control group (i.e., never contacted) (wave 5: M_treat_ = 2.23, M_control_ = 2.20, t = −0.48; wave 7: M_treat_ = 2.41, M_control_ = 2.41, t = 0.02; wave 8: M_treat_ = 2.37, M_control_ = 2.40, t = 0.37). In wave 6 (age 15), the treatment group reported slightly higher internalizing symptoms compared to the control group (wave 6: M_treat_ = 2.50, M_control_ = 2.34, t = −2.33). However, the difference is minimal, and does not take into account baseline levels of internalizing symptoms or factors that may influence selection into treatment (i.e., police contact).

**Fig. 2:**
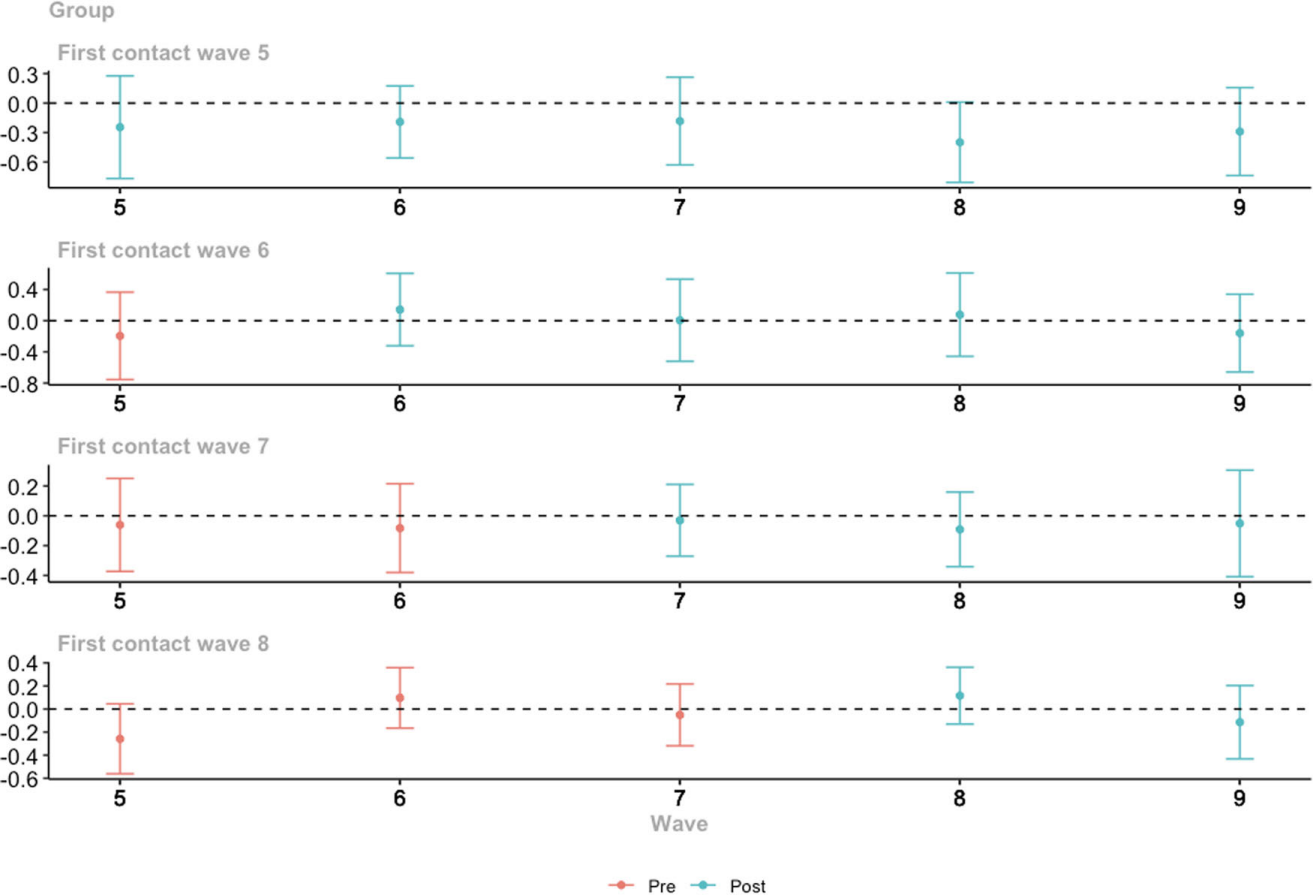
Group-time ATT results for police contact on internalizing problems

Figure 2 shows the average treatment effects for the treated population (ATT) for each group and time point (group-time), using the doubly robust estimator. The red points and confidence bands represent pre-treatment estimates, whereas the green points represent estimated treatment effects (Callaway & Sant’Anna, 2021b). Recall that the most basic interpretation of ATT reflects the difference between those who experienced their first contact (treated) and those who were never treated in the change in internalizing problems from pre- to post-treatment (i.e., before and after contact). ATT(5,5) therefore refers to the estimated difference in change in internalizing problems for the group first treated (police contact) prior to wave 5 on internalizing problems measured at wave 5 (age 13). ATT(5,6) refers to the difference in change for those first treated at wave 5 on internalizing problems at wave 6, and so on. Overall, the results show that police contact did not have a significant impact on internalizing problems at any time point. The size of these effects was also generally small, and largely negative, which is counter to expectations. For those treated in waves 6 and 8, the “immediate” or short-term estimates were positive (i.e., ATT(6,6) = 0.14, SE = 0.16, ATT(8,8) = 0.12, SE = 0.09), however, the confidence intervals overlapped zero (see Table 2). In practical terms, this suggests that the difference in change in internalizing problems for those experiencing first-time police contact in wave 6 (age 15) compared to those never treated was on average 0.14, conditional on covariates. The “pre-test” including covariates for parallel trends assumption was not significant (*p* = 0.15), suggesting that this assumption is not violated. The pre-treatment trends in Fig. 2 were not significant and for the most part hover around 0, which further suggests that the model likely does not violate the assumption, although this is only an indication and not a direct test.

**Table 2:** Difference-in-differences results for group-time ATT with covariates

	estimate	95%CI (lower)	95%CI (upper)
ATT(5,5)	−0.25 (0.18)	−0.77	0.28
ATT(5,6)	−0.19 (0.13)	−0.56	0.17
ATT(5,7)	−0.18 (0.15)	−0.63	0.26
ATT(5,8)	−0.40 (0.14)	−0.81	0.01
ATT(5,9)	−0.29 (0.15)	−0.74	0.16
ATT(6,5)	−0.19 (0.19)	−0.76	0.37
ATT(6,6)	0.14 (0.16)	−0.32	0.61
ATT(6,7)	0.01 (0.18)	−0.52	0.53
ATT(6,8)	0.08 (0.19)	−0.46	0.61
ATT(6,9)	−0.16 (0.17)	−0.66	0.34
ATT(7,5)	−0.06 (0.11)	−0.37	0.25
ATT(7,6)	−0.08 (0.10)	−0.38	0.21
ATT(7,7)	−0.03 (0.08)	−0.27	0.21
ATT(7,8)	−0.09 (0.09)	−0.34	0.16
ATT(7,9)	−0.05 (0.12)	−0.41	0.31
ATT(8,5)	−0.26 (0.10)	−0.56	0.05
ATT(8,6)	0.10 (0.09)	−0.17	0.36
ATT(8,7)	−0.05 (0.09)	−0.32	0.22
ATT(8,8)	0.12 (0.09)	−0.13	0.36
ATT(8,9)	−0.11 (0.11)	−0.43	0.20

Notes. ATT(g,t), where g represents the group (wave of first contact) and t represents the post-treatment time period (wave). Standard errors are clustered by individuals, the control group is “never treated”, and the estimation method is doubly robust. The “did” package computes ATT (g,t) using complete observations in the specific time periods and groups (treatment and control)

**Fig. 3:**
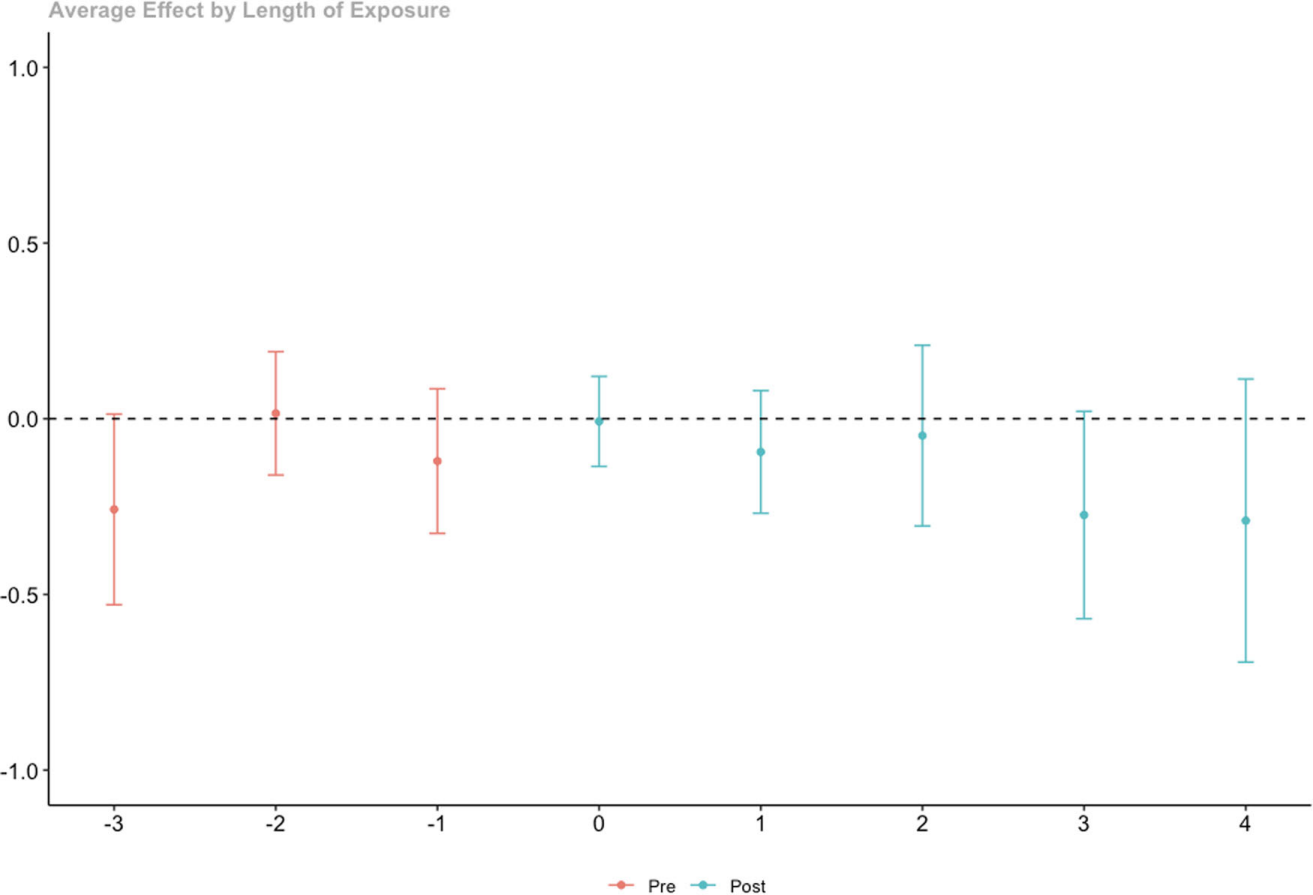
Aggregated ATT by dynamic event time for police contact on internalizing problems

Since group-time treatment effects may mask average before and after trends, estimates were aggregated using a dynamic or event-study approach. This provides ATTs in the time periods before and after the event. The results are presented in Fig. 3. The aggregated results are consisted of the group-time effects, in that they show no change, or even declines, in internalizing problems in the time periods following police contact (full results available in Supplementary Materials, Appendix B, Table B1).

**Fig. 4:**
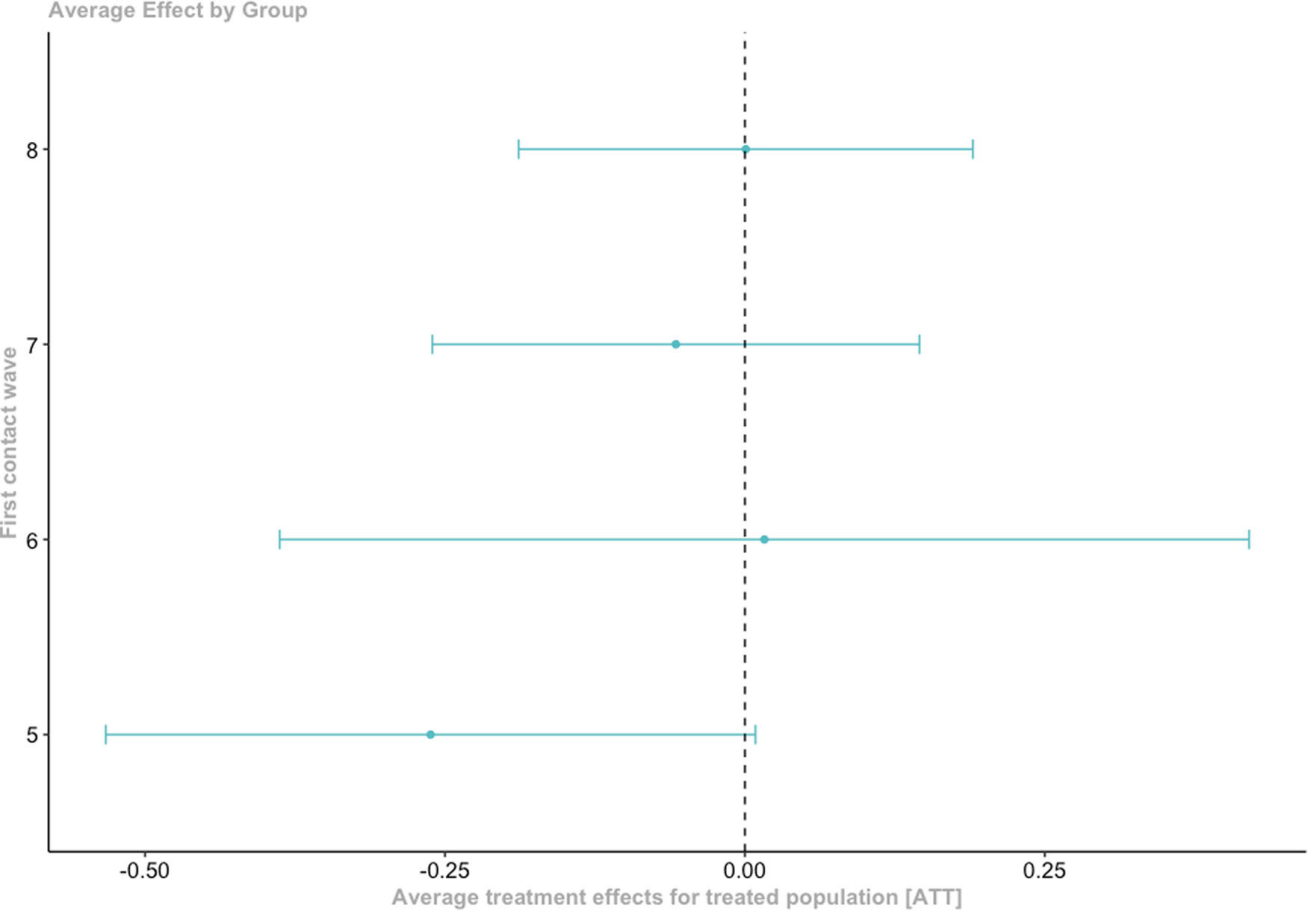
Aggregated ATT by group for police contact on internalizing problems

In addition, ATTs were aggregated by group, which shows the overall average treatment effect for those first treated at a given time point. The results are presented in Fig. 4 (full results in Supplementary Materials, Appendix B, Table B2). Figure 4 shows that the average treatment effects were null for groups 6 (first treated in wave 6, age 15) through 8 (first treated in wave 8, age 20). For those first treated in wave 5 (age 13), the ATT was negative (ATT(5) = −0.26, SE = 0.11), indicating that these participants reported an overall (slightly) lower frequency of internalizing problems compared to the never treated control group. The overall ATT across groups was −0.08 (95%CI = −0.19, 0.03), suggesting that there was no overall significant change in internalizing problems among the treated compared to those who were never treated.

### Additional analyses

Importantly, one key assumption is that the contact is experienced as “negative” in that it is likely to trigger subsequent physiological and emotional stress responses. The assumption is that contact in relation to wrongdoing (e.g., under suspicion or arrest) is experienced as negative, however, the current measure does not include self-reported perceptions of the specific contact, such as perceived unfairness of police treatment. This underlying assumption was therefore further evaluated. Specifically, the same analytical design was applied to investigate to what extent police contact, as defined by the treatment variable, was associated with more negative perceptions of police legitimacy in the short and long term. A significant effect on police legitimacy would indicate support for the assumption that the contact was experienced as “negative”, such that it had negative implications for an individual’s perceptions of fairness and trustworthiness of police Dennison & Finkeldey (2021). More information on this procedure and the full results are available in Appendix C. Independent samples t-tests showed that police contact was associated with significantly lower perceptions of police legitimacy at each wave. The group-time results showed that the ATT for group 7 (first treated in wave 7, age 17) was negative and significantly different from zero (ATT(7,7) = −0.29, 95%CI = −0.52, −0.05). The remaining group-time results were negative but not significant (see Figure C1). The aggregate dynamic results show that police contact had a significant negative effect on police legitimacy at the time of treatment (ATT(0) = −0.22, 95%CI = −0.36, −0.07). However, this effect did not last over time (see Figure C2). Aggregated by group, the overall treatment effect was ATT(average) = −0.11 (95%CI = −0.21, −0.002), however, no significant group treatment effects were found (see Figure C3). Taken together, these findings can be interpreted in support of the assumption that the police contact treatment variable had negative consequences for the perceived fairness and legitimacy of the police, and thus theoretically likely to trigger an emotional stress response.

As an additional check on the sensitivity of these results, more traditional analyses were conducted to assess whether the findings were model-specific. First, fixed effects regressions were conducted using the constructed treatment variable (first contact with police) as well as a variable indicating any contact with the police in a given wave. Since fixed effects models account for any time-invariant heterogeneity, only time-varying variables were included in the models (i.e., aggression, delinquency, low self-control, moral neutralization, peer delinquency, and victimization). Models were run with and without delinquency included. The results presented in Appendix D (Table D1) show that the change from no contact to first (or any) contact with police was associated with a small, non-significant increase in internalizing problems by about 0.04. Excluding delinquency, the change from no to any contact with police was about 0.05. The coefficient for any contact is near the conventional threshold of significance at *p* = 0.043, but given multiple tests this may be due to chance. All other time-varying variables were significantly related to internalizing problems. Second, another common approach to estimating effects of contact on mental health outcomes was used: propensity score matching. Specifically, propensity score analyses were used to match and compare individuals who reported contact with the police (in relation to wrongdoing) at a given wave and those who did not report contact (control). Here differences were examined between those reporting first police contact at a given wave with those who were never contacted, as well as differences between those who reported any police contact at a given wave with those who did not report contact in the same wave. In the latter set of analyses, participants were not excluded that had experienced police contact in wave 4, as the interest was if any police contact in relation to wrongdoing had an effect on internalizing problems. Further details on this procedure, along with the full results, are available in Appendix D. In short, no significant differences were found between treatment (first or any police contact) and control (never or no police contact) groups in the level of internalizing problems at any wave.^2^

In addition, given the potential for multicollinearity between some covariates (i.e., delinquency, self-control, moral neutralization, peer delinquency), the DiD analyses were estimated without these variables. These variables may also influence aggression, a key potential confound, and so excluding these variables may offer a more ‘clean’ assessment of the effect of first contact. Additionally, all models were estimated without covariates. The results, presented in Appendix E, remain the same for both models excluding these covariates and models without covariates. Notably, the “pre-test” for the parallel trends assumption was 0.08 in the model without covariates. While this is not significant by conventional standards, the pre-trends do seem to fluctuate in a way that may indicate a violation of the assumption (see Figures E1–E3, Appendix E).

Next, in order to assess whether the results may be different for the subscales of anxiety and depression, analyses were conducted using these separate subscales as outcomes. The group-time and aggregated treatment effects of police contact were not significant for either of the subscales of anxiety or depression (results presented in Appendix F).

There may also be important sex differences in the effects of police contact on internalizing symptoms. Increases in internalizing symptoms during adolescence are more likely to be observed among females rather than males (Patton et al., 2014), whereas young men are more likely to come in contact with police during adolescence (Mcara & Mcvie, 2005). Separate DiD analyses were therefore conducted for male and female youth. As a first step, these models were estimated without covariates. The pre-test for parallel trends was not significant for both models (*p* = 0.477 for males, *p* = 0.376 for females). Similar to the main analyses, the results were non-significant (see Appendix G), however, note that the number of treated in each group is small so the results should be treated with caution.

Finally, given the divergence in contact and perceived treatment by ethnic minorities and those with a migration background (Geller, 2021), the results were evaluated to examine variation between participants with and without a migrant background in the same manner as with sex. In line with the main analyses, the results remained largely non-significant (see Appendix H). The ATT for group 5 (first treated at age 13) at wave 9 (age 24) among those with a migrant background was negative and significant (−0.461), however, the confidence intervals were close to overlapping zero (95%CI: −0.89, −0.03). Again, dividing up the groups means that the number of treated in each sub-group is small, so the results should be considered with caution.

## Discussion

There has been a lack of research on how police contact influences within-individual changes in mental health, particularly outside the United States. This study aimed to evaluate the effect of negative police contact (i.e., being suspected or arrested) on internalizing problems among Swiss youth in the short- and long term. Using extensions to difference-in-difference techniques to estimate the average treatment effects of first contact compared to those who have never reported contact, no significant differences in internalizing problems were found at any age or time period. Additional analyses using fixed effects regressions and propensity score matching comparing those reporting any contact at a given age to those who did not report contact also generally showed no significant differences in internalizing problems. These results are inconsistent with previous research that has found police contact to increase a range of mental health issues following contact (e.g., Geller et al., 2014). Several reasons, including cross-national differences in contexts of policing, social policy, and juvenile justice may explain why police contact did not necessarily influence internalizing problems in the Swiss context.

First, the vast majority of previous research has been conducted in the United States, where policing has a long history of aggressive and racially disparate practices (Braga et al., 2019), with disproportionately high rates of fatal police violence compared to other developed countries (Hirschfield, 2023). This may create conditions for more stressful interactions, which may be more likely to activate the negative affective responses that impact mental health outcomes among youth. However, it is important to note that many of these studies focus on between-persons differences and cannot fully account for potential threats to internal validity, including selection or reverse causality (Petersen et al., 2023). Within-person studies tend to show much smaller effects (e.g., Del Toro et al., 2019). This study’s estimates are not too different in size from these latter results, although the (non-significant) direction of the relationship in the findings fluctuated between positive and negative.

Nevertheless, the consistently null results may in part reflect the differing contexts of policing and police-citizen interactions in Switzerland compared to the United States. In 2019, Switzerland recorded one fatal police shooting (rate of 0.116 per 1 million) compared to 1021 (rate of 3.080 per 1 million) in the US in 2020 (Hirschfield, 2023). Nearly 37% of young people in the sample reported contact at least once across all waves, which is notably higher than the percentage of personal contacts reported in other studies (e.g., 19% in the US, Geller, 2021; 14.77% in UK, Jackson et al., 2021). However, these encounters may still be comparatively less stress-inducing than youth encounters with the police in the United States. These differences may be a reflection of dosage, wherein especially minority youth in the US may experience a higher intensity of contact, and particularly intrusive and forceful contact, compared to Switzerland and similar European countries. In addition, the consequences of contact may vary across contexts. In particular, the stress of contact due to wrongdoing may differ depending on the punitiveness of the juvenile justice system. The Swiss juvenile justice system focuses more on diversion, which means that youth may expect to face less severe (and perhaps less stress-inducing) consequences from contact with the police related to wrongdoing compared to youth in more punitive juvenile justice contexts. These differences may alternatively reflect the particular historical context of racially disparate practices in US policing (Bell, 2017). This suggests that the effects of police contact on mental health found in the US are not necessarily generalizable to other contexts.

In addition, there may be important differences in access to social and health “safety nets” among youth experiencing internalizing problems in Zurich compared to the more vulnerable populations captured in several US-based studies (e.g., using the Fragile Families and Child Wellbeing dataset, Jackson et al., 2022). While studies show that the gap between mental health needs and treatment is relatively similar between Switzerland (Werlen et al., 2020) and the United States (up to 50% or more, Han et al., 2017), the barriers to accessing care differ between countries. For example, in the US, insurance is one of the strongest correlates of receiving treatment, and the inability to afford the cost of treatment is one of the most common structural barriers to accessing needed care (Walker et al., 2015). By contrast, it is mandatory to have health insurance in Switzerland, with lower overall costs and expenses covered for those experiencing financial hardship (Schneeberger & Schwartz, 2018). Future research should pay closer attention to how access to treatment and care may condition to what extent adverse experiences and stress responses ultimately lead to more persistent internalizing problems.

This study shows that the effect of police contact on internalizing problems may be more heterogeneous and context-dependent than expected based on previous research, of which most have been conducted using US samples. In addition, studies that rely on cross-sectional data where police contact is measured once retrospectively, and/or those that cannot account for changes in the outcome and time-varying confounders may potentially overestimate the effect of police contact on internalizing problems and other mental health outcomes. More research is needed to examine the possible effects (null or otherwise) of police contact on youth outside of the US to determine when and under what circumstances police-initiated contact leads to worsening mental health outcomes. It is possible that the existing body of evidence is a reflection of the particular context of aggressive policing in the US, but to what extent is not known without further comparative research. In a recent systematic review on the effects of police stops, 82% (*n* = 33) of studies were conducted in the US, and only 17.5% (*n* = 7) in Europe, of which 5 were conducted in the UK (Petersen et al., 2023). The authors found no studies outside of the US and Europe that fit their inclusion criteria. More studies are needed in contexts of policing reflecting different levels of public trust, (non-)aggressive policing tactics, health care, and juvenile justice policies. More broadly, well-designed comparative studies can help test the boundaries of expectations about police contact and health, and to what extent there is a need to develop more context-sensitive theories that can better inform police practice and prevention strategies (M. Eisner, 2023). Ideally, these studies should measure both contact and mental health outcomes repeatedly over time to disentangle causal order and effects.

It is important to consider several limitations to this study. First, the focus was primarily on “negative” contact defined by police-initiated interaction in relation to wrongdoing. There was not a measure of how the participant perceived this interaction in relation to fairness or intrusiveness. Previous research has found that intrusive and unfair contact is more likely to generate negative feelings of distress and mental health problems (McFarland et al., 2019). While this study was able to establish that the contact negatively impacted perceptions of police fairness and legitimacy, it is not certain how the participants perceived police behavior within the specific interaction. In addition, the measure of contact was relatively broad, as the aim was to capture any negative police-initiated contact in the time period prior to the survey. These contacts may be in response to a variety of deviant behaviors, however, most reasons for contact were relatively minor (e.g., fare dodging, underage drinking, drug use). These types of contacts may not be perceived as serious enough to produce persistent feelings of distress and negative affect that can lead to worse mental health outcomes. Nevertheless, these are not uncommon circumstances under which youth may come in contact with the police. For example, young people are more likely to commit fare evasion (Cools et al., 2018), and there is evidence in the US to suggest that transit police may unfairly target and ticket ethnic minority passengers compared to white passengers (Carter & Johnson, 2021). In addition, the null result remained when using the more restricted and arguably more serious life event measure of police contact. Future research should examine more closely in which contexts youth are coming into contact with the police, and to what extent this may impact the underlying mechanisms of distress and negative affect.

Second, while the analysis includes some attempts to assess whether the parallel trends assumption was violated, the assumption can never be directly tested. It is still possible that there may be unobserved differences in the trend following treatment between the treated and untreated groups. The conditional parallel trends assumption can account for this issue to some extent, but also depends on the covariates available and included in the analyses. In addition, although several measures were taken to account for the “cascading” effects of externalizing problems and prior delinquency (Murray et al., 2020), it is not entirely possible with retrospective measures to disentangle the potential effects of prior antisocial behavior or (suspected) wrongdoing from the police contact itself. Similarly, the reliability of causal estimates based on propensity score matching depends on the quality of covariates included in the matching process, or whether there is no omitted variables violating the conditional independence assumption (Huntington-Klein, 2022). While a wide range of theoretically-relevant covariates were included that may explain both selection and trends, there may still be unobserved and unmeasured differences that were not accounted for in the matching process. These problems of potential or unaccounted confounds are persistent in research examining the effects of police contact (Petersen et al., 2023). Quasi-experimental approaches are useful for approximating randomization in treatment, but it is important to remember that there are still challenges in achieving “as-good-as-random” using observational data. The current study suggests that there is little evidence for causal, or even simple bivariate, effects of police contact on internalizing problems among youth in Zurich. Future studies aiming to assess causal effects should be careful to account for all theoretically-relevant covariates where possible when assessing assumptions and to use multiple quasi-experimental methods to ensure the results are not model-dependent.

Third, it was not possible to investigate the underlying mechanisms that connect police contact to internalizing problems, such as stress responses and emotional dysregulation. Studies using ecological momentary assessment show that police contact was associated with next-day psychological distress (Del Toro et al., 2022), however, more research is needed to understand when and how this distress may persist over time to affect longer-term health outcomes.

Finally, it is important to emphasize that the period between contact and reporting internalizing problems could be quite large (i.e., potentially ranging from months to a maximum 3 years prior) with the precise timing unknown. This is a problem that is not uncommon to research on police contact and health outcomes (Petersen et al., 2023). The potentially large time frame in between means that one cannot rule out alternative factors or events that might occur in between contact and the time of the survey that may account for these (null) effects. In particular, in relation to policing and arrests, a number of subsequent factors, such as social, emotional, or financial losses due to arrest, and/or consequent convictions and punishments may emerge as a result of contact, which may account for the relationship between negative police contact and mental health issues. More research is needed to understand the immediate effects that police contact has on individuals (see e.g., Del Toro et al., 2022), how the effects of these momentary encounters unfold over time from days to years, and to what extent other intermediate factors may contribute to (or confound) the relationship between contact and mental health.

## Conclusion

Research on the relationship between police contact and mental health outcomes has been primarily conducted in the US, often using designs that cannot account for baseline measures of mental health. This study suggests that negative police contact, defined by police-initiated contact in relation to wrongdoing, does not lead to changes in internalizing problems among youth in Zurich, Switzerland. This is possibly due to differences in the context of policing between Switzerland and the United States, the source of most prior studies. Specifically, the policies and policing context in Switzerland mean that youth may be subject to less intrusive interactions compared to those in the United States, resulting in less stressful or negative affective responses that can generate internalizing problems. It may also be that the historical legacy of aggressive and discriminatory practices in the US generates more pervasive feelings of stress related to (personal and vicarious) interactions compared to Switzerland and similar countries. In any case, more research is needed, especially outside the US, to understand under which circumstances, contexts, and life stages contact is most likely to generate stress that can cause increases in internalizing problems and other mental health issues.

## Supplementary information

Appendix

## Footnotes

1 This is generally in line with broader social surveys conducted in Switzerland, such as the European Social Survey, which showed that 42.9% of respondents in Switzerland reported being approached, stopped, or contacted by the police between 2008 and 2020 (Staubli, 2016).

2 Additionally, effects were estimated using only the general retrospective measure of police contact as part of a major life events scale. This variable referred to police contact in relation to wrongdoing, but not specific delinquent or criminal acts. As part of the life events scale, this measure may arguably capture more serious contact compared to the contact reported in relation to self-reported (usually minor) delinquency. Notably, the number of first-time contacts measured using this variable is relatively lower than the combined treatment variable (treated w5 = 51, w6 = 67, w7 = 43, w8 = 52). Given this small sample size, the effects were estimated using only sex to minimize loss of information due to potentially missing data. The test for parallel trends was not significant (*p* = 0.0978). The results remained the same (see Appendix B, Figures B4-B6).
