# Supplementary material for: Assessing the Effect of First-time Police Contact on Internalizing Problems Among Youth in Zurich, Switzerland: A Quasi-experimental Analysis
Source: J Youth Adolesc. 2024 Apr 25;53(8):1711–27. doi: 10.1007/s10964-024-01986-9 (PMC11226482; doi:10.1007/s10964-024-01986-9)
Supplement: Supplementary file 1 — Appendix [file 10964_2024_1986_MOESM1_ESM.docx]

Supplementary Materials for “Assessing the effect of first-time police contact on internalizing problems among youth in Zurich, Switzerland: A quasi-experimental analysis”

[Appendix A – Construction of treatment variable and selection of covariates 2](#_Toc161322401)

[Police contact treatment variable 2](#_Toc161322402)

[Construction and selection of covariates 2](#_Toc161322403)

[Appendix B – Full results for DiD analyses 5](#_Toc161322404)

[Appendix C – Evaluating assumptions about “negative” police contact 10](#_Toc161322405)

[Appendix D – Additional fixed effects and propensity score analyses 14](#_Toc161322406)

[Appendix E – Results without covariates and excluding certain covariates 20](#_Toc161322407)

[Appendix F – Disaggregated results for anxiety and depression 26](#_Toc161322408)

[Appendix G – Disaggregated results for males and females 32](#_Toc161322409)

[Appendix H – Disaggregated results by migrant background 38](#_Toc161322410)

[References 44](#_Toc161322411)

# Appendix A – Construction of treatment variable and selection of covariates

## Police contact treatment variable

Several variables were used to construct the treatment variable in order to capture potential contacts due to wrongdoing. The first was a general retrospective measure of police contact in relation to wrongdoing in the years prior to the survey. This measure was part of a life history calendar, which asked about the prevalence of major life events in waves 5 through 9 (i.e., “You got reported to the police and were questioned by them”). Notably, this indicates that the respondent was questioned, but does not necessarily mean that they were arrested or charged for the offense. Being stopped or questioned by the police was associated with worse mental health outcomes among youth in the UK (Jackson et al., 2021). The second was in relation to the specific self-reported deviant behavior items. If respondents indicated that they had engaged in a particular behavior in the past 12 months, a follow-up question asked how many times they had to deal with the police because of that behavior. An indicator of the prevalence of police contact was constructed in relation to these specific behaviors. The number of delinquent behaviors included in this scale changed over time, in part because older youth were assumed to become engaged in a greater range of delinquent behaviors. However, since the primary interest was in creating a general measure of any “treatment” (police contact in relation to wrongdoing) that respondents experienced, items were not excluded regardless of (in)consistent measurement over time. These questions were asked in waves 5 through 9.

The third additional variable asked respondents whether they had any contact with the police in the past two years. These items were only included in waves 4 through 7. Respondents could then indicate the nature of the contact, including acting as a witness or victim, being stopped by the police, in relation to several possible deviant acts, or some other reason. In wave 4, the follow-up questions only referred to possible deviant acts, whereas in the following waves other possible reasons for contact were included. The focus here was on whether or not respondents had contact in relation to the list of possible deviant acts, and does not include those who have reported contact as a witness or victim. Taken together, the first reported negative contact with the police was coded based on the above set of variables.

## Construction and selection of covariates

The selection of covariates was based on prior research on within-individual variation in internalizing problems, as well as research on factors that influence police contact (Dennison & Finkeldey, 2021; Wiley et al., 2013). For example, previous work that has used propensity score matching to account for selection into police arrest and contact has shown that there are significant differences in low self-control, moral neutralizations, and peer delinquency that should be accounted for in the matching process (Jackson et al., 2020). Measures of aggression and deviant behavior were also included, which have been shown to correlate with internalizing problems over time throughout adolescence (Murray et al., 2020), and often end up “converting” to internalizing problems later in life (Copeland et al., 2009). Externalizing behaviors, especially engagement in criminal behavior, are also likely to increase the likelihood of contact with the police (Wiley et al., 2013). Since certain stressful life events could also influence internalizing problems over time (McLaughlin & Hatzenbuehler, 2009), a measure of serious victimization that has occurred in the 12 months prior to the survey was included.

Ideally, covariates were selected that were measured in all six waves, however, given the changing nature of the longitudinal panel throughout different life stages, this was not always possible as scales were removed and/or items were changed to fit the developmental context. Where scales were included in all waves, but items were changed, the scales were restricted to include only items that were measured across all waves. Where theoretically-relevant scales were removed, meaning it was not possible to use covariates that were measured across all waves, an overall average score was created based on available waves. This means that, while not ideal, variables relevant to explaining selection or internalizing problems were not excluded from the matching process.

**Table A1**. Descriptive statistics and bivariate correlations for variables in wave 5 (age 13)

| Variable | *M* | *SD* | 1 | 2 | 3 | 4 | 5 | 6 | 7 | 8 | 9 | 10 | 11 | 12 |
| --- | --- | --- | --- | --- | --- | --- | --- | --- | --- | --- | --- | --- | --- | --- |
| 1. First reported police contact (wave 5) | 0.09 | 0.29 |  |  |  |  |  |  |  |  |  |  |  |  |
| 2. Male | 0.51 | 0.5 | .13** |  |  |  |  |  |  |  |  |  |  |  |
| 3. Migrant background | 0.49 | 0.5 | 0.00 | -0.03 |  |  |  |  |  |  |  |  |  |  |
| 4. SES | 46.23 | 19.18 | 0.01 | 0.03 | -.45** |  |  |  |  |  |  |  |  |  |
| 5. Self-reported delinquency | 1.34 | 1.41 | .23** | .21** | -.06* | 0 |  |  |  |  |  |  |  |  |
| 6. Aggression | 1.75 | 0.58 | .25** | .27** | .08** | -.11** | .46** |  |  |  |  |  |  |  |
| 7. Serious victimization | 0.21 | 0.4 | .11** | .12** | -0.03 | -.07* | .27** | .21** |  |  |  |  |  |  |
| 8. Internalizing problems | 2.2 | 0.73 | 0.01 | -.29** | -.09** | .07* | .11** | .17** | .15** |  |  |  |  |  |
| 9. Unstructured leisure time (average) | 1.32 | 0.4 | .20** | .32** | .07* | -.09** | .43** | .49** | .15** | -0.05 |  |  |  |  |
| 10. Stressful life events (average) | 0.61 | 0.43 | 0.03 | -0.05 | .15** | -.11** | .10** | .08** | .11** | .07* | .11** |  |  |  |
| 11. Low self-control | 2.2 | 0.47 | .18** | .13** | -0.05 | 0.01 | .41** | .52** | .16** | .16** | .40** | .07* |  |  |
| 12. Moral neutralization | 1.95 | 0.59 | .20** | .28** | .14** | -.16** | .38** | .62** | .14** | -0.02 | .46** | .07* | .50** |  |
| 13. Delinquent peers | 0.14 | 0.2 | .20** | .19** | -0.05 | 0.04 | .52** | .37** | .13** | 0.03 | .40** | .09** | .38** | .35** |

# Appendix B – Full results for DiD analyses

**Table B1**. Difference-in-differences results aggregated by dynamic/event-study ATT with covariates

| ATT | estimate | 95%CI (lower) | 95%CI (upper) |
| --- | --- | --- | --- |
| ATT(-3) | -0.26 (0.10) | -0.53 | 0.01 |
| ATT(-2) | 0.02 (0.07) | -0.16 | 0.19 |
| ATT(-1) | -0.12 (0.08) | -0.33 | 0.09 |
| ATT(0) | -0.01 (0.05) | -0.14 | 0.12 |
| ATT(1) | -0.09 (0.07) | -0.27 | 0.08 |
| ATT(2) | -0.05 (0.10) | -0.31 | 0.21 |
| ATT(3) | -0.27 (0.11) | -0.57 | 0.02 |
| ATT(4) | -0.29 (0.16) | -0.69 | 0.11 |

Notes. ATT(t), where t represents the time period before (t-1) and after (t+1) the treatment event. Standard errors are clustered by individuals, the control group is “never treated”, and the estimation method is doubly robust.

**Table B2**. Difference-in-differences results aggregated by group ATT with covariates

|  | estimate | 95%CI (lower) | 95%CI (upper) |
| --- | --- | --- | --- |
| ATT(Average) | -0.08 (0.06) | -0.19 | 0.03 |
| ATT(5) | -0.26 (0.12) | -0.54 | 0.02 |
| ATT(6) | 0.02 (0.17) | -0.39 | 0.42 |
| ATT(7) | -0.06 (0.08) | -0.24 | 0.12 |
| ATT(8) | 0.00 (0.09) | -0.20 | 0.21 |

Notes. ATT(g), where g represents the group, or time of first contact (e.g., 5=first contact at wave 5, age 13). Standard errors are clustered by individuals, the control group is “never treated”, and the estimation method is doubly robust.


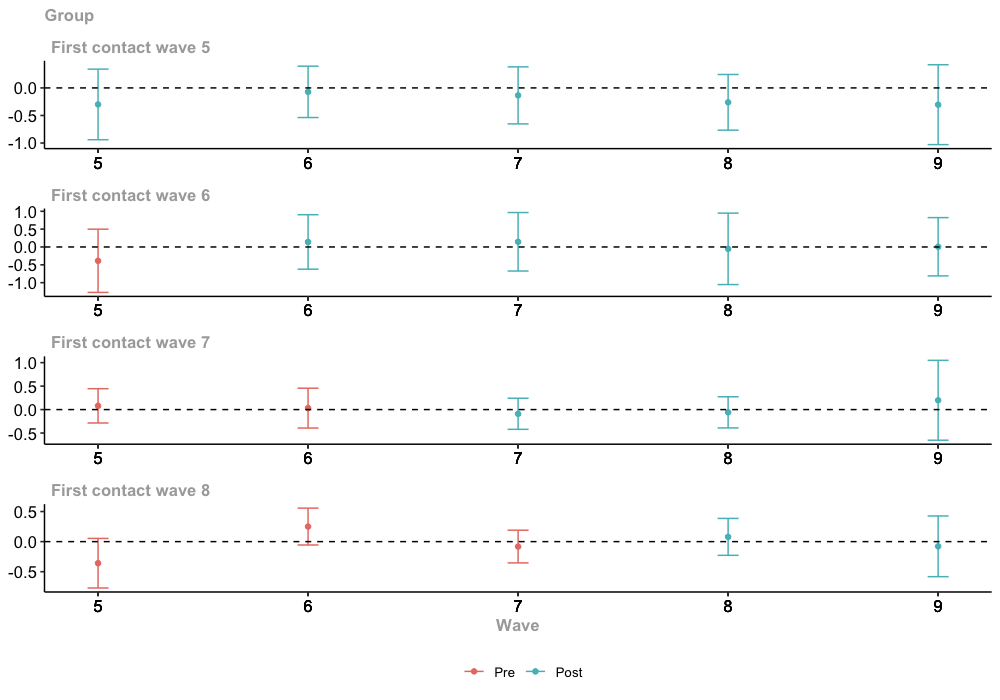


**Figure B4**. Group-time ATT for internalizing problems using only the life events measure of police contact (with sex as covariate)


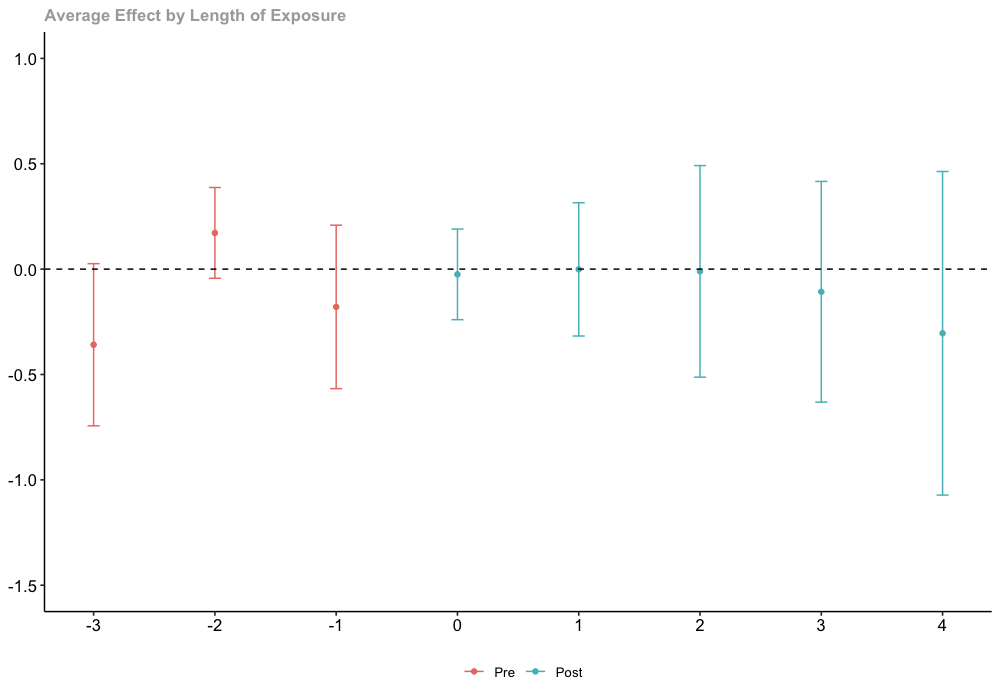


**Figure B5**. Results aggregated by dynamic/event-study ATT for internalizing problems using only the life events measure of police contact (with sex as covariate)


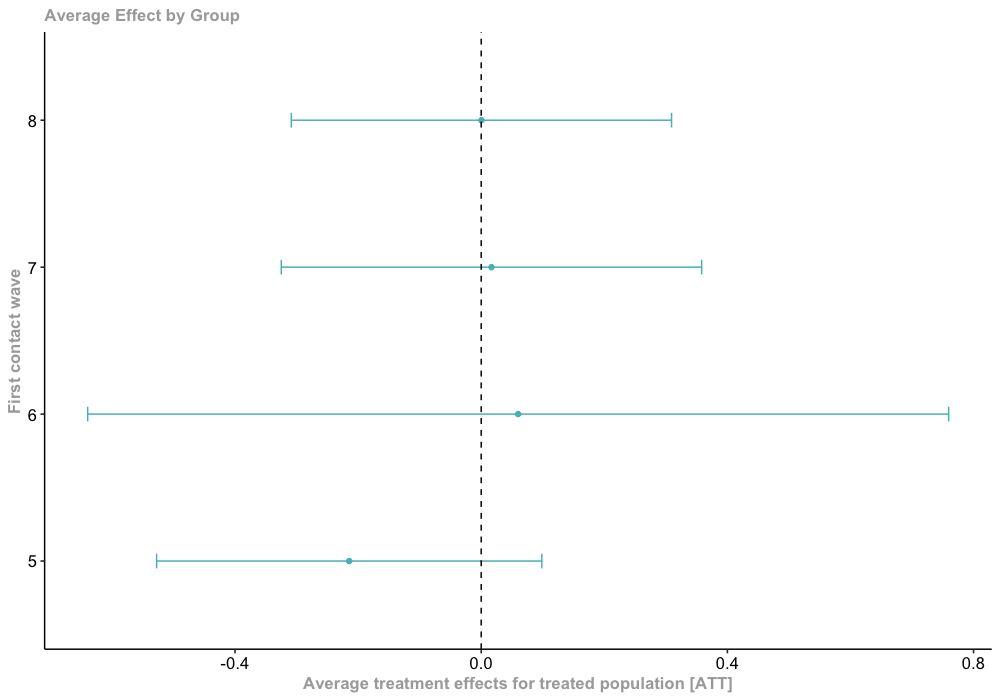


**Figure B3**. Results aggregated by group ATT for internalizing problems using only the life events measure of police contact (with sex as covariate)

# Appendix C – Evaluating assumptions about “negative” police contact

In order to evaluate the assumption that our measure of police contact was experienced as negative, we used three items that capture elements of procedural fairness (i.e., the quality of treatment and respect), fairness decision-making (i.e., applying the rules consistently), and police effectiveness (i.e., doing their jobs well). Respondents indicated agreement on a four-point, Likert-type scale ranging from “fully untrue” to “fully true”. The items were measured at ages 15 (alpha=0.82), 17 (alpha=0.87), 20 (alpha=0.84), and 24 (alpha=0.86).

In order to establish a baseline of untreated respondents, we excluded those who reported contact in waves 4 (age 11), 5 (age 15), and 6 (age 15), as well as wave 9 (age 24). This means that we assess short-term treatment effects at wave 7 (age 17), and long-term effects at wave 8 (age 20). We first conducted independent samples t-tests to evaluate whether police contact at each wave (including wave 6) was associated with differences in police legitimacy. The results showed that individuals who reported police contact also reported significantly lower perceptions of police legitimacy compared to those who never reported contact (wave 6: M_treatment_=2.19, M_control_=2.74, *t*=5.69; wave 7: M_treatment_=2.20, M_control_=2.59, *t*=3.24; wave 8: M_treatment_=2.24, M_control_=2.60, *t*=3.19).

Next, we applied the same difference-in-differences procedure to police legitimacy. Covariates were not included in the model, however the “pre-test” for the parallel trends assumption was not significant (p=0.40).


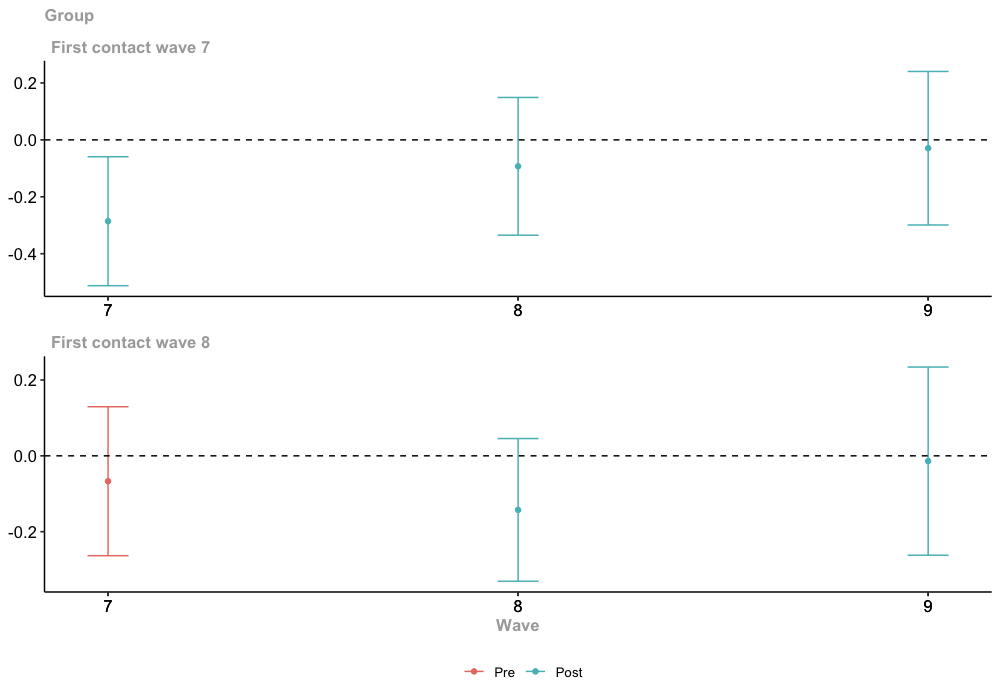


**Figure C1**. Group-time ATT for police legitimacy


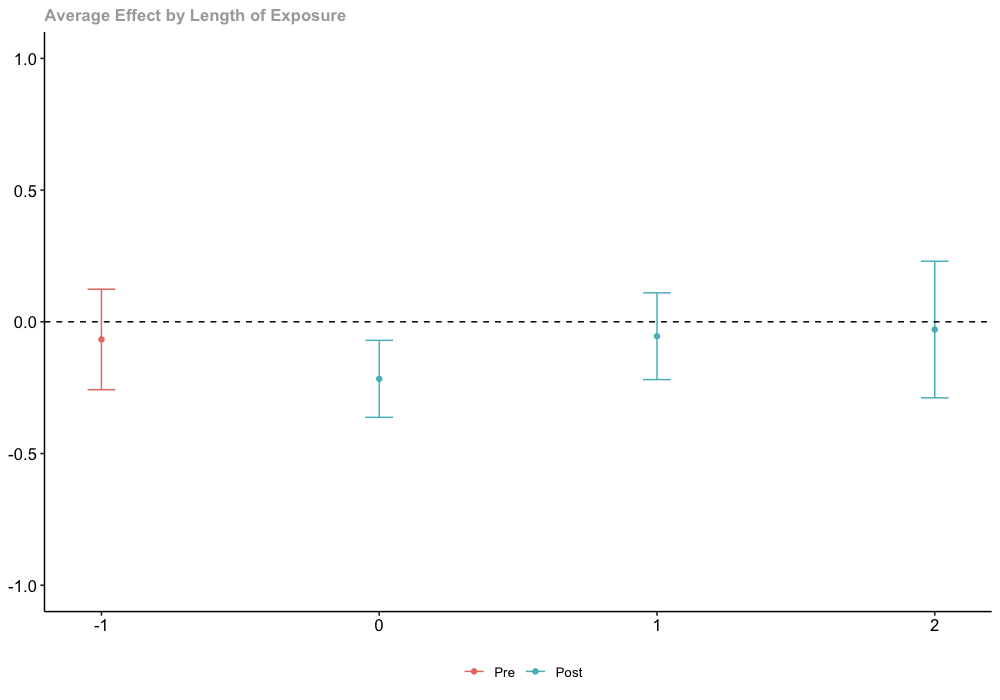


**Figure C2**. Results aggregated by dynamic/event-study ATT for police legitimacy


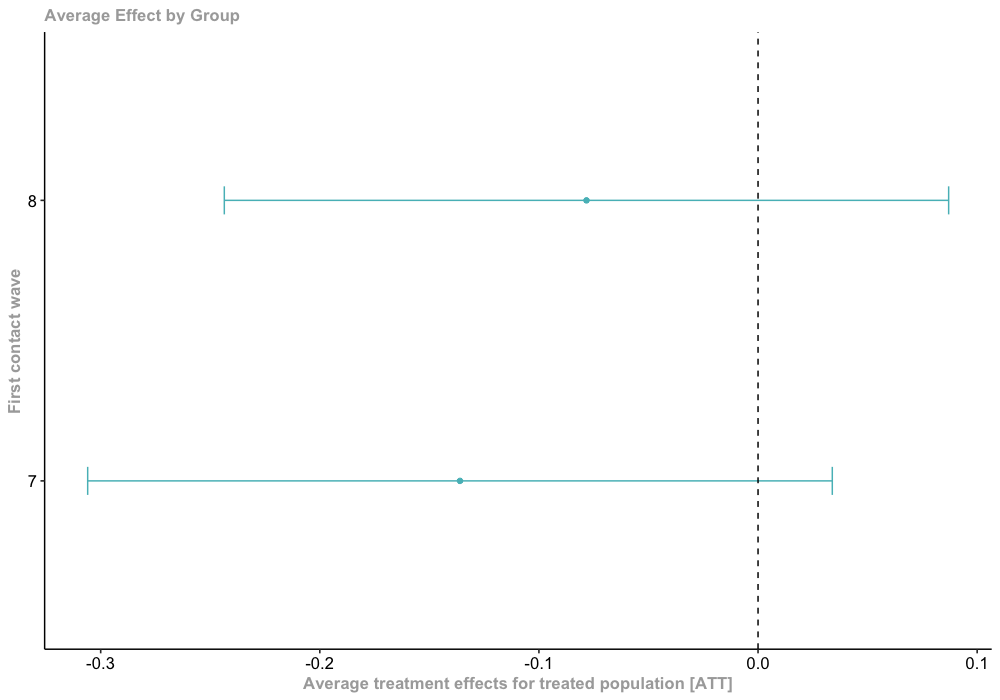


**Figure C3**. Results aggregated by group ATT for police legitimacy

# Appendix D – Additional fixed effects and propensity score analyses

First, fixed effects analyses were conducted using both the treatment variable, in which respondents are coded as 1 when they have reported their first contact with police and in all following waves (no reported contact=0), and a variable indicating whether the respondent has reported any contact in relation to wrongdoing in a given wave. Time-varying covariates from the main model were included. Heteroskedacity-robust standard errors are presented (HC1). Analyses were conducted using the *plm* package in R Studio (Croissant & Millo, 2008). The results are presented in Table D1.

**Table D1**. Fixed effects regression of police contact on internalizing problems

|  | Model 1  (First contact FE) | Model 2  (Any contact FE) | Model 3  (First contact FE excl. delinquency) | Model 4  (Any contact FE excl. delinquency) |
| --- | --- | --- | --- | --- |
| First-reported police contact | 0.046 (0.036) |  | 0.062 (0.036) |  |
|  | [-0.025, 0.117] |  | [-0.008, 0.133] |  |
| Any police contact |  | 0.044 (0.027) |  | 0.053 (0.026)* |
|  |  | [-0.009, 0.097] |  | [0.002, 0.105] |
| Aggression | 0.183 (0.030)*** | 0.181 (0.029)*** | 0.187 (0.029)*** | 0.183 (0.029)*** |
|  | [0.125, 0.241] | [0.123, 0.238] | [0.130, 0.244] | [0.127, 0.240] |
| Delinquency | 0.021 (0.010)* | 0.021 (0.010)* |  |  |
|  | [0.002, 0.041] | [0.002, 0.040] |  |  |
| Low self-control | 0.123 (0.032)*** | 0.121 (0.032)*** | 0.128 (0.031)*** | 0.126 (0.031)*** |
|  | [0.060, 0.185] | [0.058, 0.183] | [0.066, 0.189] | [0.064, 0.187] |
| Moral neutralization | -0.075 (0.028)** | -0.076 (0.028)** | -0.079 (0.027)** | -0.081 (0.027)** |
|  | [-0.129, -0.021] | [-0.130, -0.022] | [-0.132, -0.026] | [-0.135, -0.028] |
| Peer delinquency | 0.319 (0.046)*** | 0.323 (0.045)*** | 0.344 (0.043)*** | 0.350 (0.042)*** |
|  | [0.229, 0.409] | [0.235, 0.411] | [0.259, 0.429] | [0.267, 0.433] |
| Victimization | 0.088 (0.030)** | 0.087 (0.030)** | 0.095 (0.030)** | 0.093 (0.030)** |
|  | [0.028, 0.147] | [0.027, 0.146] | [0.037, 0.154] | [0.035, 0.151] |
| N | 4692 | 4692 | 4766 | 4766 |
| R2 | 0.052 | 0.052 | 0.05 | 0.05 |
| Std.Errors | HC1 | HC1 | HC1 | HC1 |

Note. Standard errors in parentheses, 95% confidence intervals in brackets. Analyses were conducted using waves 5 (age 13) to 8 (age 20).

Second, the covariates outlined in the methods section were used, measured at the previous wave or as an average, in a logistic regression model to estimate the probability of treatment assignment, or the propensity score (Austin, 2011). We then used 1:1 nearest neighbor matching without replacement to construct matched treatment and control pairs for analysis. When the matching procedure is successful, the standardized mean difference between treatment and control should be less than 0.1 (Austin, 2011). If this was not achieved, we included those covariates in the weighted regression model when estimating treatment effects (Nguyen et al., 2017). The ATT was the marginal effect of police contact experienced prior to the survey on internalizing behavior measured in the same wave. The outcome regression and calculation of marginal effects included the propensity score matching weights, with cluster-robust standard errors (Ho et al., 2011). Analyses are conducted on the listwise sample using *MatchIt* in R (Ho et al., 2011).

The matching results for wave 5 show that there were no substantial imbalances on covariates between those in the treated and control groups (see Table D2). There matching process created 117 matched pairs for analysis. The ATT shown in Table D3 was 0.12 (95%CI=-0.05, 0.30). For wave 6, there were several imbalances on the covariates remaining after the matching process, including aggression, prior internalizing problems, and criminal leisure activities. These covariates were therefore included in the weighted regression when estimating the effects and marginal effects on the outcome. There were 220 matched pairs in wave 6. The ATT was 0.07 (95%CI=-0.06, 0.20). In wave 7, there were also imbalances remaining after matching, which were included in the estimation of the regression and marginal effects. The ATT was -0.03 (95%CI=-0.17, 0.12). In wave 8, again we included a number of covariates where imbalanced remained. The ATT was 0.06 (95%CI=-0.06, 0.19).

**Table D2**. Means and standardized mean differences between unmatched and matched pairs for waves 5 through 8 (any reported police contact)

|  |  | Unmatched |  |  | Matched |  |
| --- | --- | --- | --- | --- | --- | --- |
| Wave 5 | Treated (M) | Control (M) | SMD | Treated (M) | Control (M) | SMD |
| Male | 1.70 | 1.47 | 0.50 | 0.70 | 0.73 | -0.06 |
| Migrant | 1.50 | 1.42 | 0.14 | 1.50 | 1.48 | 0.03 |
| SES | 46.68 | 48.92 | -0.11 | 46.68 | 45.18 | 0.07 |
| Delinquency (w4) | 1.40 | 0.84 | 0.37 | 1.40 | 1.32 | 0.05 |
| Aggression (w4) | 1.73 | 1.51 | 0.43 | 1.73 | 1.76 | -0.05 |
| Victimization (w4) | 0.40 | 0.29 | 0.22 | 0.40 | 0.38 | 0.03 |
| Internalizing problems (w4) | 2.08 | 2.02 | 0.08 | 2.08 | 2.05 | 0.04 |
| Criminal leisure (avg) | 1.53 | 1.27 | 0.53 | 1.53 | 1.54 | -0.02 |
| Stressful life events (avg) | 0.66 | 0.59 | 0.16 | 0.66 | 0.69 | -0.06 |
| Low self-control (w4) | 2.16 | 1.91 | 0.49 | 2.16 | 2.13 | 0.06 |
| Moral neutralization (w4) | 1.75 | 1.59 | 0.31 | 1.75 | 1.73 | 0.05 |
| Delinquent peers (w4) | 0.09 | 0.04 | 0.31 | 0.09 | 0.08 | 0.09 |
| N | 117 | 759 |  | 117 | 117 |  |
|  |  | Unmatched |  |  | Matched |  |
| Wave 6 | Treated (M) | Control (M) | SMD | Treated (M) | Control (M) | SMD |
| Male | 0.58 | 0.49 | 0.18 | 0.58 | 0.55 | 0.07 |
| Migrant | 1.47 | 1.48 | -0.02 | 1.47 | 1.45 | 0.03 |
| SES | 45.08 | 46.59 | -0.07 | 45.08 | 44.30 | 0.04 |
| Delinquency (w5) | 2.01 | 1.19 | 0.46 | 2.01 | 1.86 | 0.08 |
| Aggression (w5) | 2.01 | 1.66 | 0.49 | 2.01 | 1.93 | 0.11 |
| Victimization (w5) | 0.29 | 0.17 | 0.25 | 0.29 | 0.27 | 0.03 |
| Internalizing problems (w5) | 2.23 | 2.17 | 0.09 | 2.23 | 2.30 | -0.11 |
| Criminal leisure (avg) | 1.55 | 1.25 | 0.64 | 1.55 | 1.49 | 0.13 |
| Stressful life events (avg) | 0.65 | 0.57 | 0.19 | 0.65 | 0.62 | 0.08 |
| Low self-control (w5) | 2.43 | 2.13 | 0.65 | 2.43 | 2.42 | 0.02 |
| Moral neutralization (w5) | 2.15 | 1.88 | 0.43 | 2.15 | 2.12 | 0.05 |
| Delinquent peers (w5) | 0.24 | 0.11 | 0.48 | 0.24 | 0.24 | 0.00 |
| N | 220 | 902 |  | 220 | 220 |  |
|  |  | Unmatched |  |  | Matched |  |
| Wave 7 | Treated (M) | Control (M) | SMD | Treated (M) | Control (M) | SMD |
| Male | 0.65 | 0.46 | 0.40 | 0.65 | 0.63 | 0.04 |
| Migrant | 1.43 | 1.48 | -0.12 | 1.43 | 1.48 | -0.11 |
| SES | 48.52 | 46.35 | 0.12 | 48.52 | 47.35 | 0.06 |
| Delinquency (w6) | 2.73 | 1.36 | 0.73 | 2.73 | 2.31 | 0.22 |
| Aggression (w6) | 1.91 | 1.60 | 0.50 | 1.91 | 1.84 | 0.10 |
| Victimization (w6) | 0.23 | 0.11 | 0.29 | 0.23 | 0.21 | 0.04 |
| Internalizing problems (w6) | 2.39 | 2.35 | 0.05 | 2.39 | 2.37 | 0.03 |
| Criminal leisure (avg) | 1.52 | 1.23 | 0.62 | 1.52 | 1.46 | 0.13 |
| Stressful life events (avg) | 0.66 | 0.57 | 0.21 | 0.66 | 0.65 | 0.03 |
| Low self-control (w6) | 2.46 | 2.21 | 0.61 | 2.46 | 2.45 | 0.01 |
| Moral neutralization (w6) | 2.17 | 1.86 | 0.53 | 2.17 | 2.15 | 0.04 |
| Delinquent peers (w6) | 0.43 | 0.29 | 0.55 | 0.43 | 0.41 | 0.10 |
| N | 235 | 874 |  | 235 | 235 |  |
|  |  | Unmatched |  |  | Matched |  |
| Wave 8 | Treated (M) | Control (M) | SMD | Treated (M) | Control (M) | SMD |
| Male | 0.67 | 0.41 | 0.56 | 0.67 | 0.62 | 0.12 |
| Migrant | 1.45 | 1.46 | -0.01 | 1.45 | 1.43 | 0.05 |
| SES | 47.53 | 47.75 | -0.01 | 47.53 | 48.45 | -0.05 |
| Delinquency (w7) | 2.45 | 1.32 | 0.70 | 2.45 | 2.06 | 0.24 |
| Aggression (w7) | 1.72 | 1.48 | 0.48 | 1.72 | 1.62 | 0.20 |
| Victimization (w7) | 0.13 | 0.06 | 0.20 | 0.13 | 0.11 | 0.05 |
| Internalizing problems (w7) | 2.33 | 2.47 | -0.18 | 2.33 | 2.40 | -0.09 |
| Criminal leisure (avg) | 1.47 | 1.21 | 0.64 | 1.47 | 1.37 | 0.25 |
| Stressful life events (avg) | 0.64 | 0.56 | 0.19 | 0.64 | 0.63 | 0.04 |
| Low self-control (w7) | 2.37 | 2.13 | 0.58 | 2.37 | 2.34 | 0.08 |
| Moral neutralization (w7) | 1.98 | 1.69 | 0.48 | 1.98 | 1.86 | 0.20 |
| Delinquent peers (w7) | 0.50 | 0.39 | 0.44 | 0.50 | 0.49 | 0.03 |
| N | 251 | 675 |  | 251 | 251 |  |
| Note. SMD=standardized mean difference; M=mean. | | |  |  |  |  |

**Table D3**. Estimated ATT for police contact on internalizing problems following propensity score matching (any reported police contact)

| Wave | ATT (SE) | 95%CI lower | 95%CI upper | N |
| --- | --- | --- | --- | --- |
| Wave 5 | 0.12 (0.09) | -0.05 | 0.30 | 234 |
| Wave 6 | 0.07 (0.06) | -0.06 | 0.20 | 440 |
| Wave 7 | -0.03 (0.08) | -0.18 | 0.12 | 470 |
| Wave 8 | 0.06 (0.06) | -0.06 | 0.19 | 502 |
| Note. ATT estimates are marginal effects with cluster-robust standard errors. | | | | |

In addition, to mirror our main analyses, we used propensity score matching to assess mean differences in internalizing symptoms between youth who reported their first police contact in a given wave and those who have never reported contact with the police. Following the same processes outlined above, the results also show no significant differences between treated and control groups at any wave. The results of the matching process and ATT are presented in Tables D4 and D5, respectively.

**Table D4**. Means and standardized mean differences between unmatched and matched pairs for waves 5 through 8 (first reported police contact)

|  |  | Unmatched |  |  | Matched |  |
| --- | --- | --- | --- | --- | --- | --- |
| Wave 5 | Treated (M) | Control (M) | SMD | Treated (M) | Control (M) | SMD |
| Male | 0.69 | 0.48 | 0.44 | 0.69 | 0.69 | 0.00 |
| Migrant | 1.51 | 1.43 | 0.17 | 1.51 | 1.48 | 0.06 |
| SES | 47.03 | 48.89 | -0.09 | 47.03 | 48.91 | -0.09 |
| Delinquency (w4) | 1.32 | 0.87 | 0.32 | 1.32 | 1.32 | 0.00 |
| Aggression (w4) | 1.72 | 1.52 | 0.40 | 1.72 | 1.69 | 0.07 |
| Victimization (w4) | 0.39 | 0.29 | 0.19 | 0.39 | 0.32 | 0.13 |
| Internalizing problems (w4) | 2.09 | 2.02 | 0.10 | 2.09 | 2.10 | -0.01 |
| Criminal leisure (avg) | 1.50 | 1.28 | 0.46 | 1.50 | 1.48 | 0.03 |
| Stressful life events (avg) | 0.66 | 0.58 | 0.16 | 0.66 | 0.69 | -0.07 |
| Low self-control (w4) | 2.14 | 1.91 | 0.45 | 2.14 | 2.18 | -0.09 |
| Moral neutralization (w4) | 1.72 | 1.59 | 0.25 | 1.72 | 1.75 | -0.05 |
| Delinquent peers (w4) | 0.08 | 0.05 | 0.25 | 0.08 | 0.07 | 0.06 |
| N | 108 | 762 |  | 108 | 108 |  |
|  |  | Unmatched |  |  | Matched |  |
| Wave 6 | Treated (M) | Control (M) | SMD | Treated (M) | Control (M) | SMD |
| Male | 0.51 | 0.51 | 0.01 | 0.51 | 0.45 | 0.12 |
| Migrant | 1.38 | 1.48 | -0.21 | 1.38 | 1.43 | -0.10 |
| SES | 47.98 | 46.34 | 0.08 | 47.98 | 46.88 | 0.05 |
| Delinquency (w5) | 1.58 | 1.32 | 0.18 | 1.58 | 1.37 | 0.15 |
| Aggression (w5) | 1.86 | 1.71 | 0.26 | 1.86 | 1.85 | 0.02 |
| Victimization (w5) | 0.26 | 0.19 | 0.16 | 0.26 | 0.23 | 0.06 |
| Internalizing problems (w5) | 2.29 | 2.18 | 0.15 | 2.29 | 2.26 | 0.04 |
| Criminal leisure (avg) | 1.47 | 1.28 | 0.41 | 1.47 | 1.43 | 0.09 |
| Stressful life events (avg) | 0.63 | 0.58 | 0.09 | 0.63 | 0.62 | 0.01 |
| Low self-control (w5) | 2.39 | 2.17 | 0.51 | 2.39 | 2.41 | -0.05 |
| Moral neutralization (w5) | 2.03 | 1.92 | 0.18 | 2.03 | 1.98 | 0.09 |
| Delinquent peers (w5) | 0.19 | 0.13 | 0.28 | 0.19 | 0.19 | 0.03 |
| N | 120 | 982 |  | 120 | 120 |  |
|  |  | Unmatched |  |  | Matched |  |
| Wave 7 | Treated (M) | Control (M) | SMD | Treated (M) | Control (M) | SMD |
| Male | 0.66 | 0.48 | 0.37 | 0.66 | 0.60 | 0.12 |
| Migrant | 1.43 | 1.47 | -0.10 | 1.43 | 1.43 | 0.00 |
| SES | 52.08 | 46.39 | 0.31 | 52.08 | 54.55 | -0.13 |
| Delinquency (w6) | 2.16 | 1.61 | 0.34 | 2.16 | 2.20 | -0.02 |
| Aggression (w6) | 1.75 | 1.65 | 0.18 | 1.75 | 1.75 | 0.01 |
| Victimization (w6) | 0.21 | 0.13 | 0.19 | 0.21 | 0.17 | 0.09 |
| Internalizing problems (w6) | 2.39 | 2.36 | 0.05 | 2.39 | 2.52 | -0.19 |
| Criminal leisure (avg) | 1.37 | 1.28 | 0.26 | 1.37 | 1.37 | 0.02 |
| Stressful life events (avg) | 0.59 | 0.59 | 0.00 | 0.59 | 0.59 | -0.01 |
| Low self-control (w6) | 2.36 | 2.25 | 0.31 | 2.36 | 2.36 | 0.01 |
| Moral neutralization (w6) | 1.98 | 1.92 | 0.11 | 1.98 | 2.01 | -0.06 |
| Delinquent peers (w6) | 0.35 | 0.32 | 0.16 | 0.35 | 0.33 | 0.09 |
| N | 87 | 1014 |  | 87 | 87 |  |
|  |  | Unmatched |  |  | Matched |  |
| Wave 8 | Treated (M) | Control (M) | SMD | Treated (M) | Control (M) | SMD |
| Male | 0.61 | 0.47 | 0.28 | 0.61 | 0.62 | -0.03 |
| Migrant | 1.39 | 1.46 | -0.14 | 1.39 | 1.38 | 0.03 |
| SES | 50.16 | 47.48 | 0.13 | 50.16 | 48.84 | 0.06 |
| Delinquency (w7) | 1.77 | 1.61 | 0.15 | 1.77 | 1.86 | -0.09 |
| Aggression (w7) | 1.53 | 1.54 | -0.04 | 1.53 | 1.52 | 0.01 |
| Victimization (w7) | 0.09 | 0.08 | 0.06 | 0.09 | 0.12 | -0.09 |
| Internalizing problems (w7) | 2.29 | 2.44 | -0.18 | 2.29 | 2.36 | -0.08 |
| Criminal leisure (avg) | 1.29 | 1.28 | 0.03 | 1.29 | 1.33 | -0.16 |
| Stressful life events (avg) | 0.63 | 0.58 | 0.11 | 0.63 | 0.61 | 0.05 |
| Low self-control (w7) | 2.21 | 2.20 | 0.04 | 2.21 | 2.23 | -0.06 |
| Moral neutralization (w7) | 1.80 | 1.77 | 0.06 | 1.80 | 1.81 | -0.02 |
| Delinquent peers (w7) | 0.45 | 0.41 | 0.19 | 0.45 | 0.48 | -0.13 |
| N | 74 | 852 |  | 74 | 74 |  |
| Note. SMD=standardized mean difference; M=mean. | | |  |  |  |  |

**Table D5**. Estimated ATT for police contact on internalizing problems following propensity score matching (first reported police contact)

| Wave | ATT (SE) | 95%CI lower | 95%CI upper | N |
| --- | --- | --- | --- | --- |
| Wave 5 | 0.003 (0.09) | -0.18 | 0.19 | 216 |
| Wave 6 | 0.15 (0.10) | -0.04 | 0.34 | 240 |
| Wave 7 | 0.10 (0.09) | -0.07 | 0.27 | 174 |
| Wave 8 | 0.12 (0.14) | -0.15 | 0.39 | 148 |
| Note. Estimated marginal effects using cluster-robust standard errors. | | | | |

# Appendix E – Results without covariates and excluding certain covariates


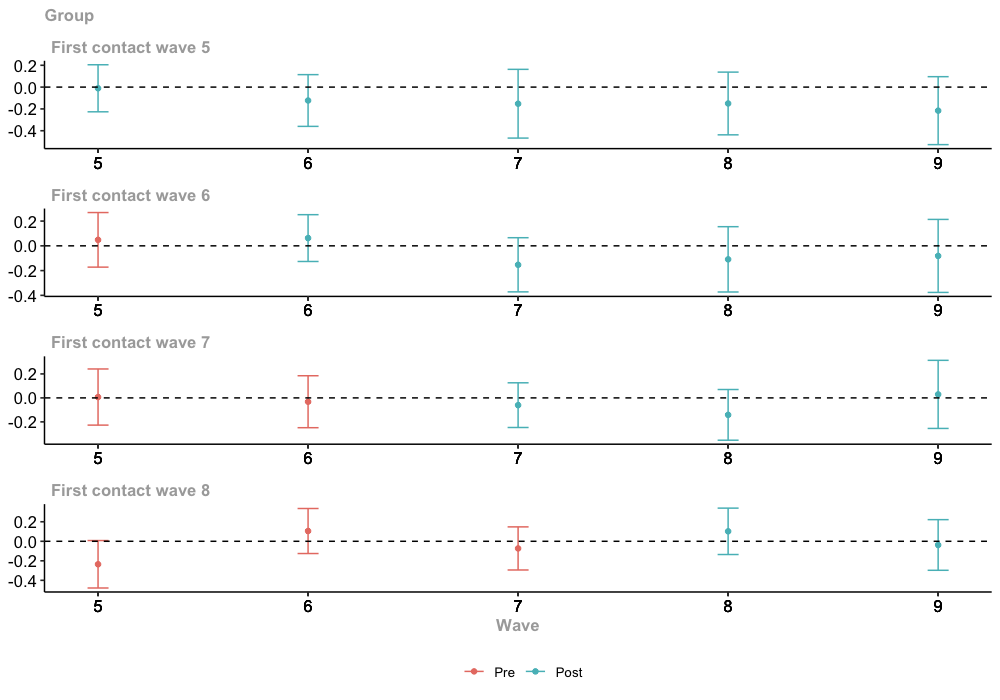


**Figure E1**. Group-time ATT for internalizing problems without covariates


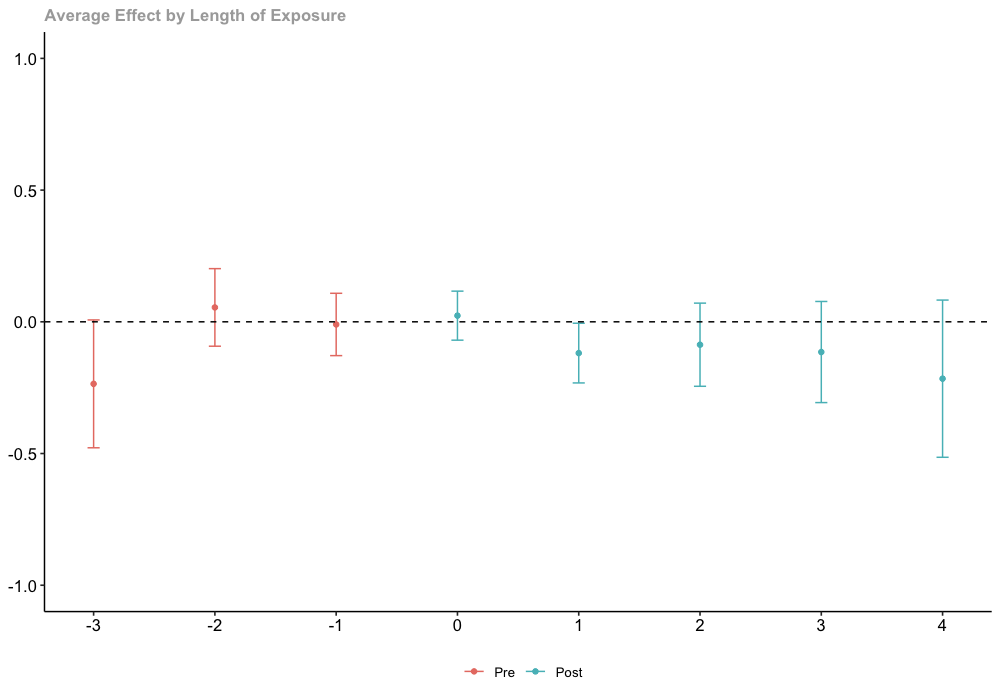


**Figure E2**. Results aggregated by dynamic/event-study ATT for internalizing problems without covariates


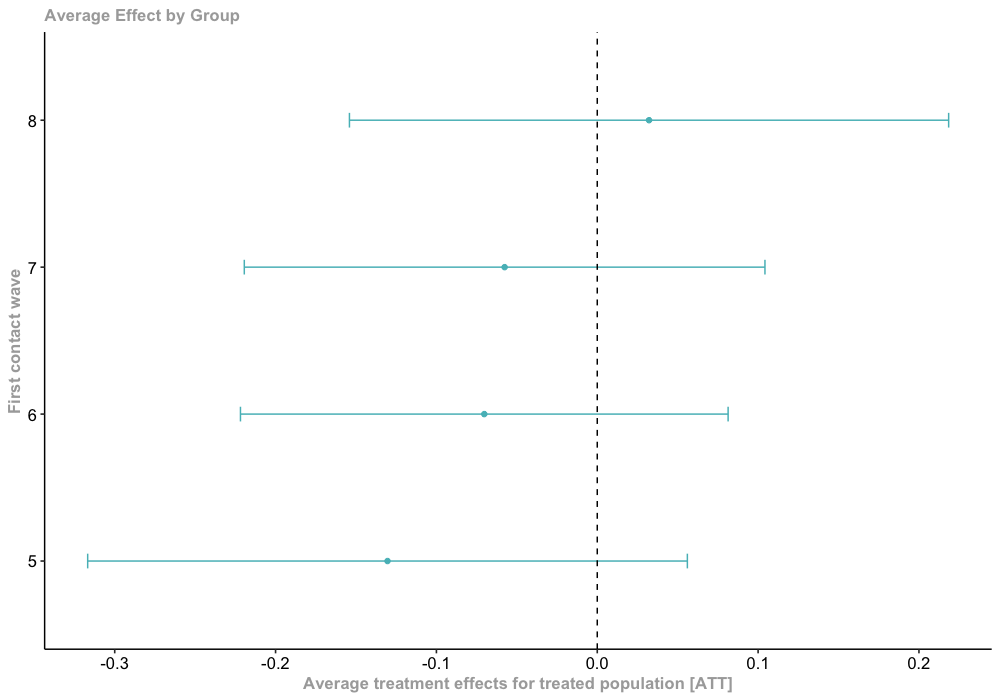


**Figure E3**. Results aggregated by group ATT for internalizing problems without covariates


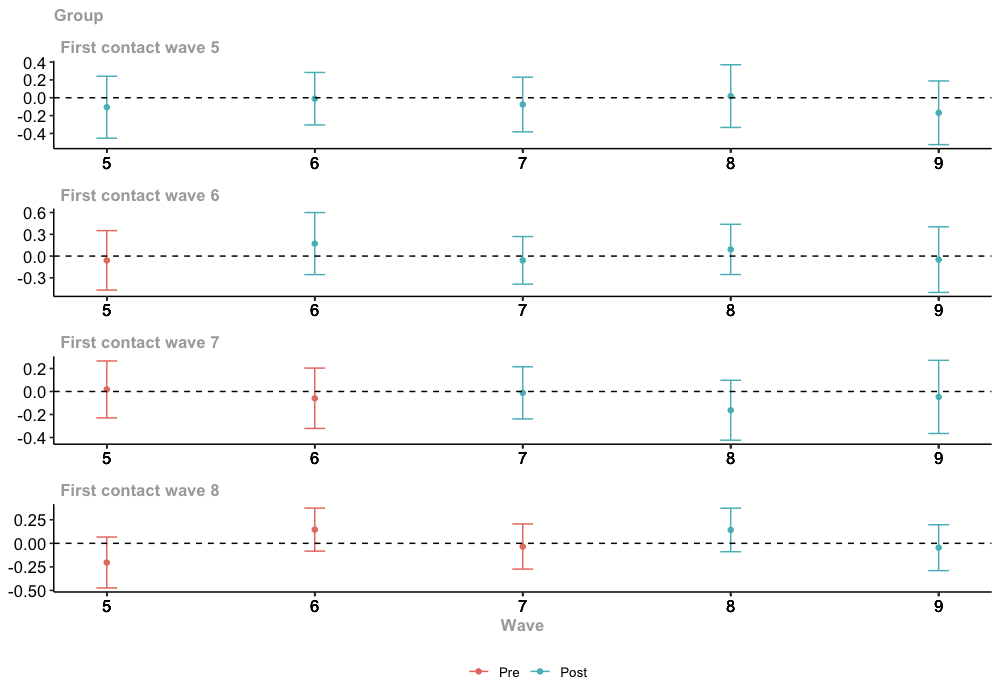


**Figure E4**. Group-time ATT for internalizing problems excluding delinquency, low self-control, moral neutralization, and peer delinquency

**
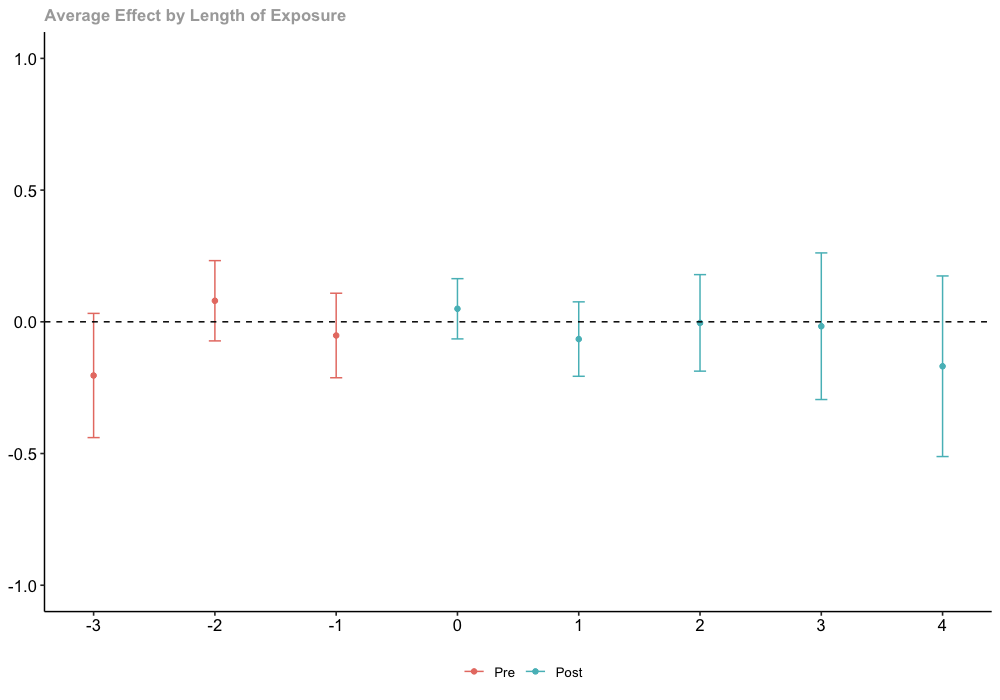
**

**Figure E5**. Results aggregated by dynamic/event-study ATT for internalizing problems excluding delinquency, low self-control, moral neutralization, and peer delinquency


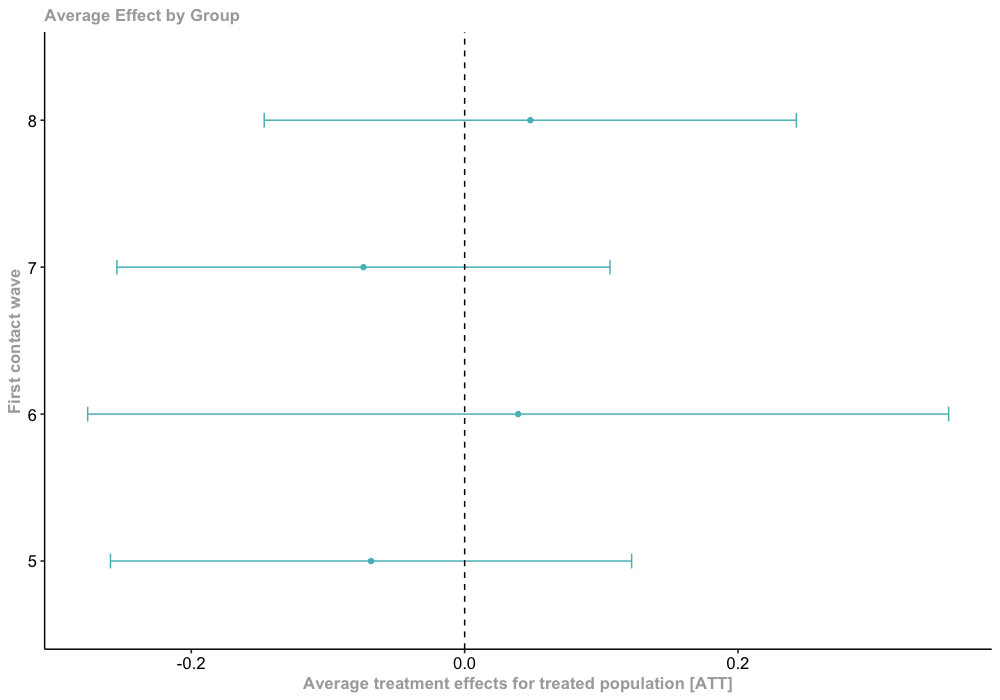


**Figure E6**. Results aggregated by group ATT for internalizing problems without covariates excluding delinquency, low self-control, moral neutralization, and peer delinquency

# Appendix F – Disaggregated results for anxiety and depression

All analyses were conducted using the same covariates and process as the main analyses. It is important to note that the reliability for each subscale, in particular anxiety in early waves was less than ideal, with alphas between 0.60 and 0.70. In addition, while the pre-test for parallel trends for depression was not significant (p=0.45), the pre-test for anxiety was borderline (p=0.049). We made small adjustments to the covariates, excluding average levels of depression and anxiety, after which the pre-test became non-significant (p=0.09).


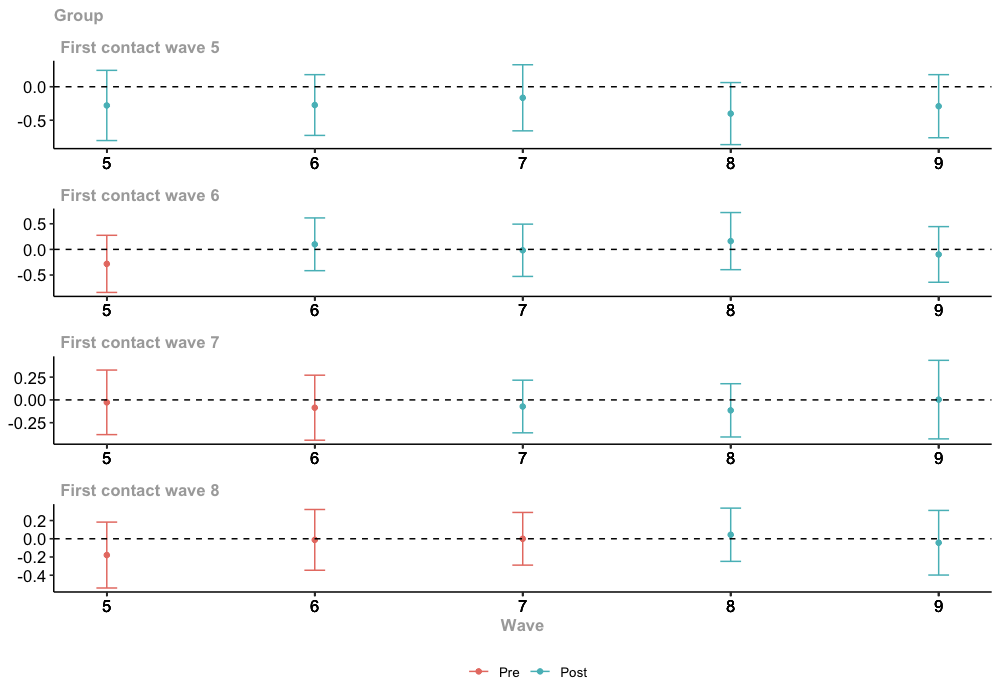


**Figure F1**. Group-time ATT for depression


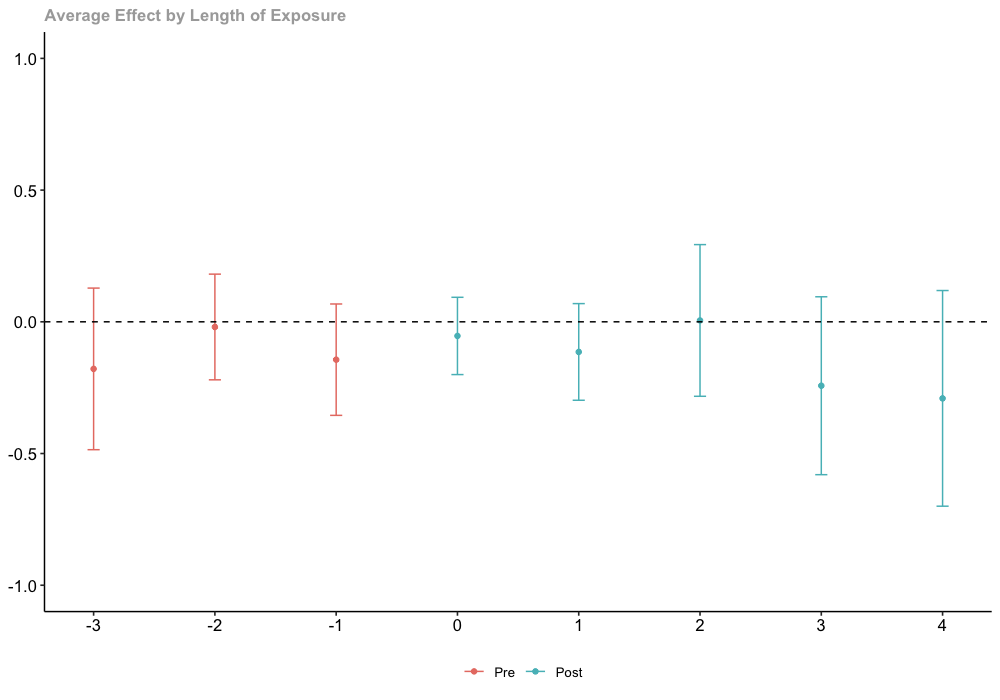


**Figure F2**. Results aggregated by dynamic/event-study ATT for depression


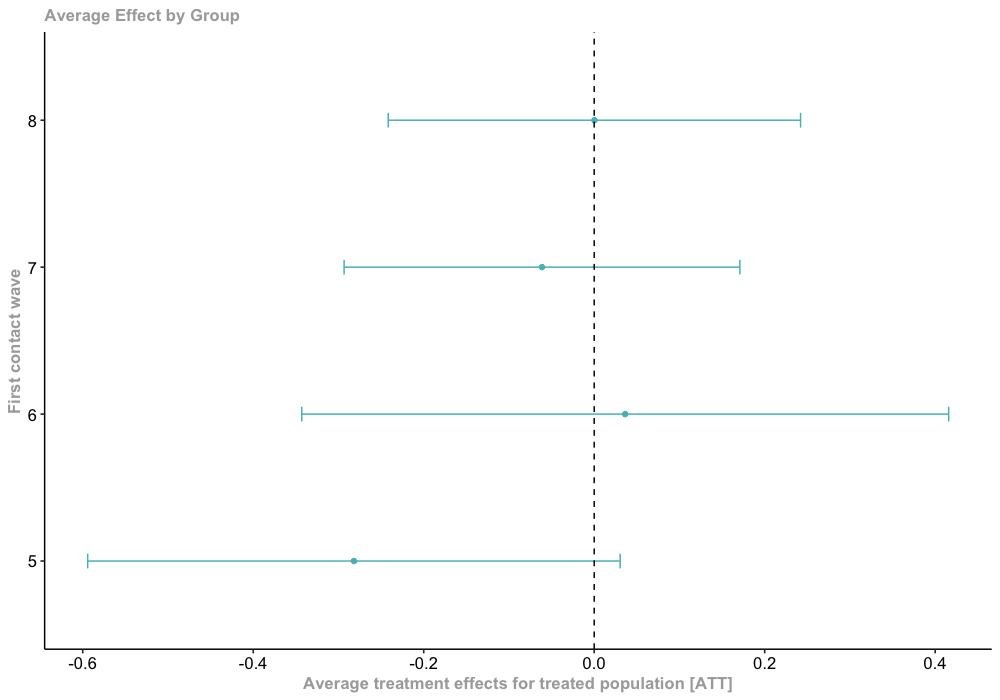


**Figure F3**. Results aggregated by group ATT for depression


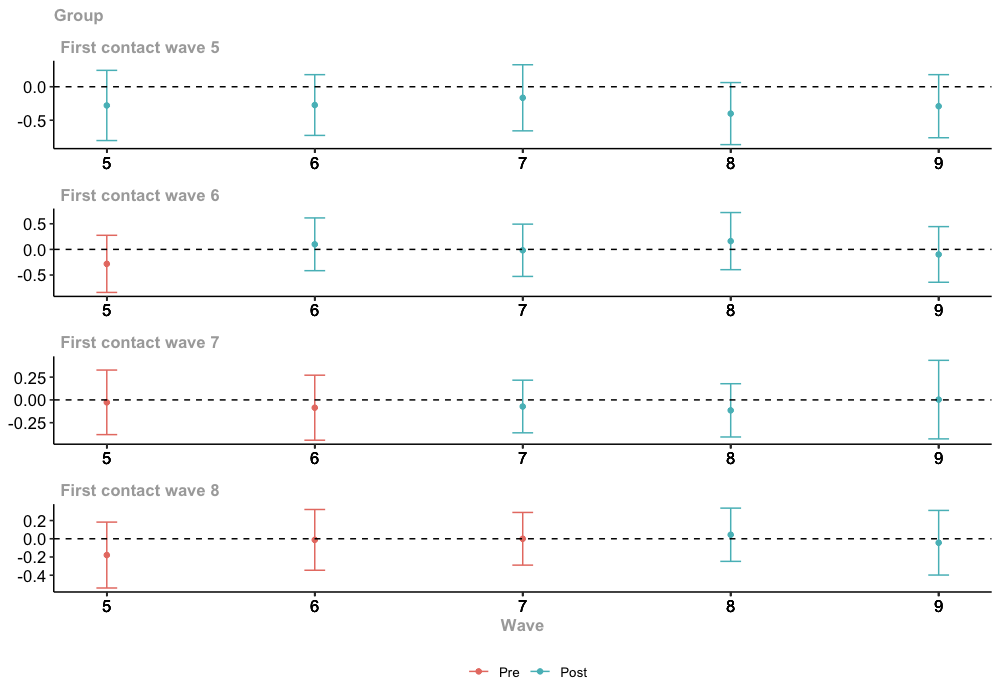


**Figure F4**. Group-time ATT for anxiety


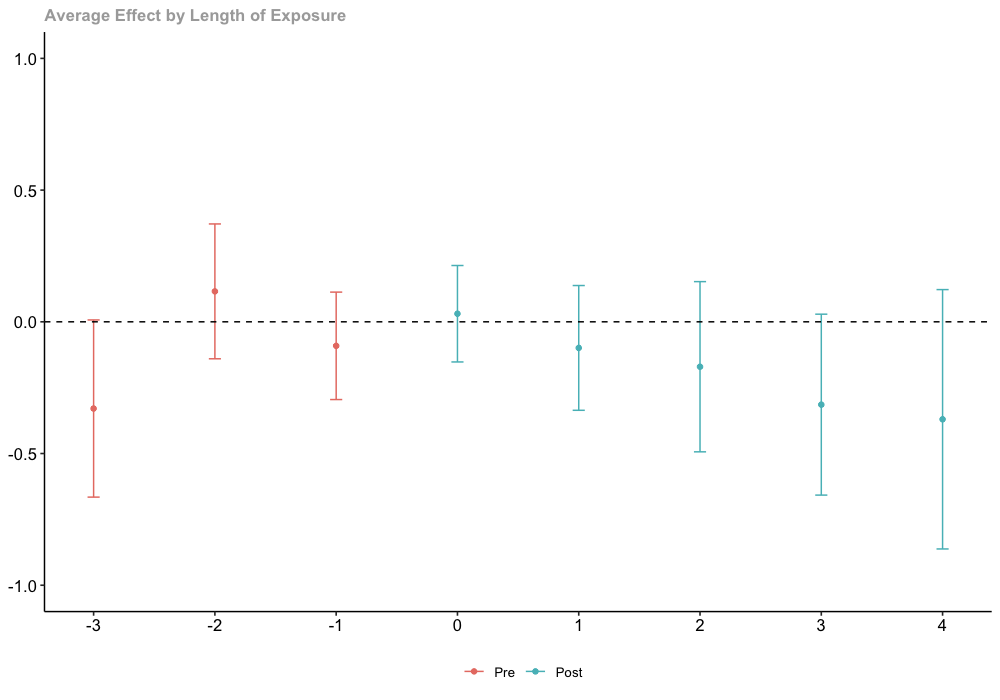


**Figure F5**. Results aggregated by dynamic/event-study ATT for anxiety


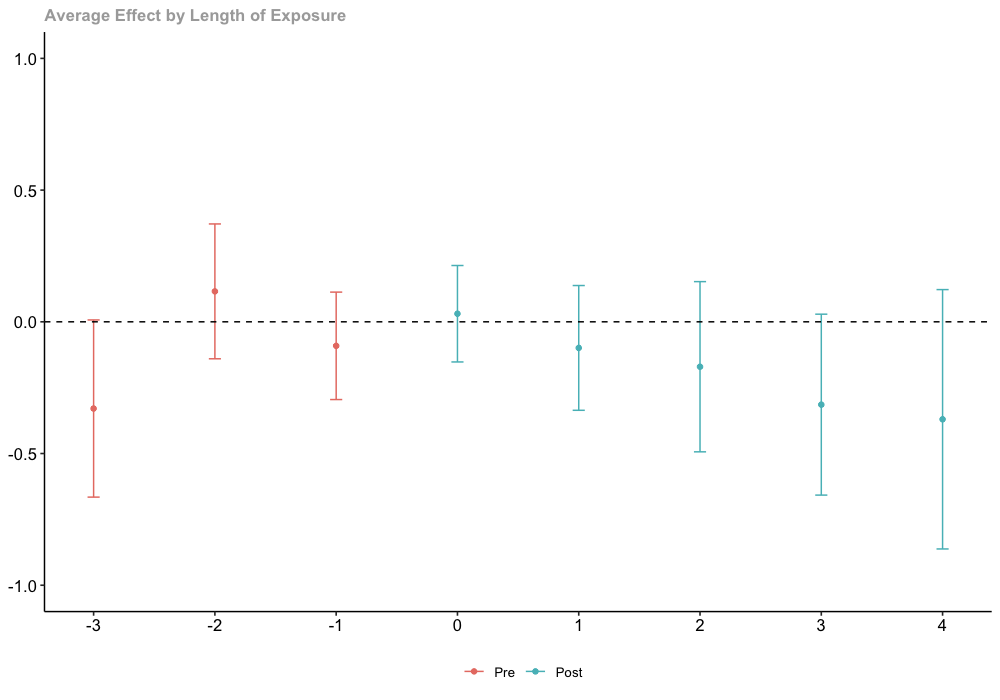


**Figure F6**. Results aggregated by group ATT for anxiety

# Appendix G – Disaggregated results for males and females

All analyses were conducted using the same process as the main analyses, but excluding covariates.


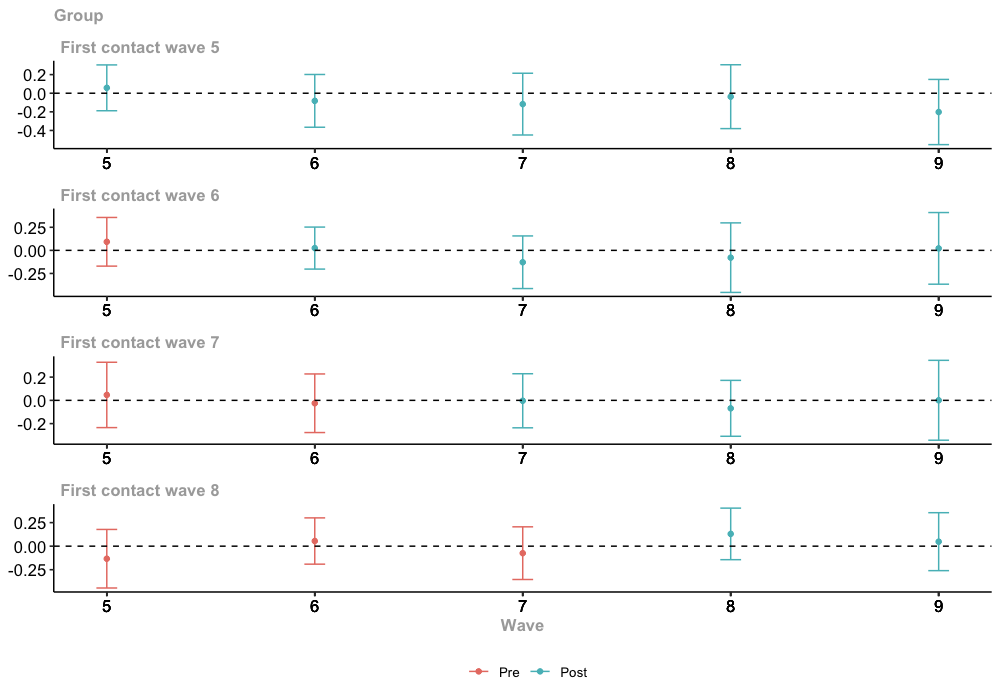


**Figure G1**. Group-time ATT for males


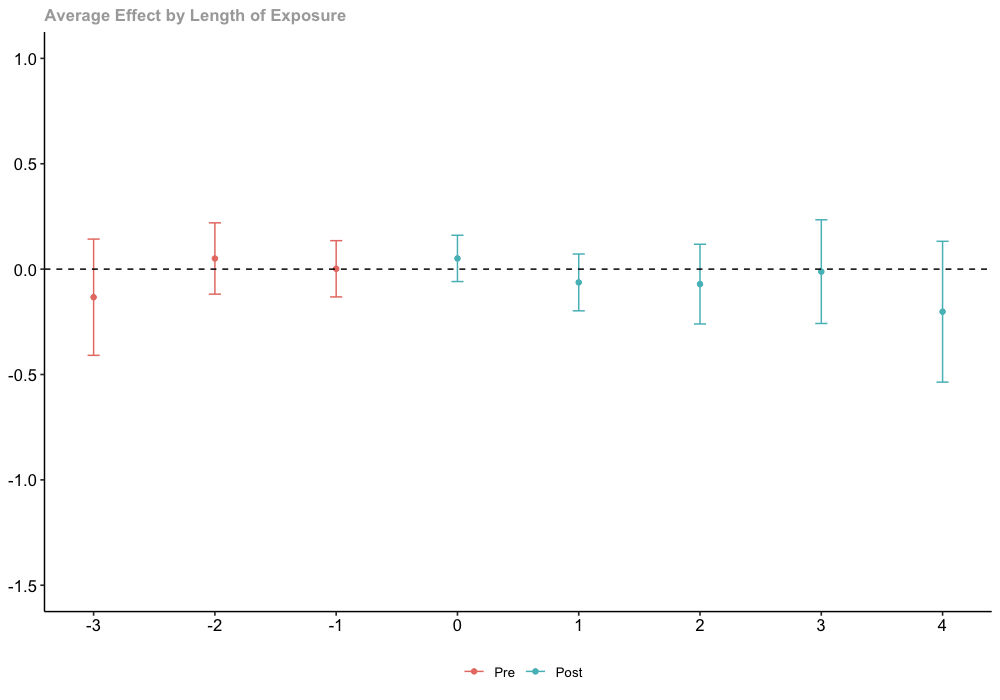


**Figure G2**. Results aggregated by dynamic/event-study ATT for males


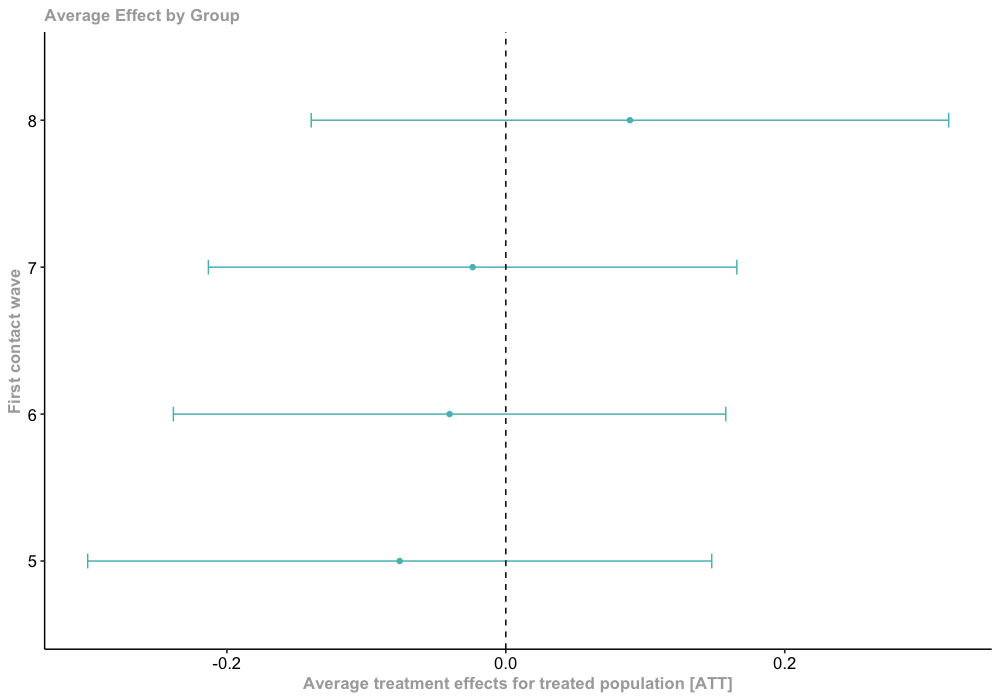


**Figure G3**. Results aggregated by group ATT for males


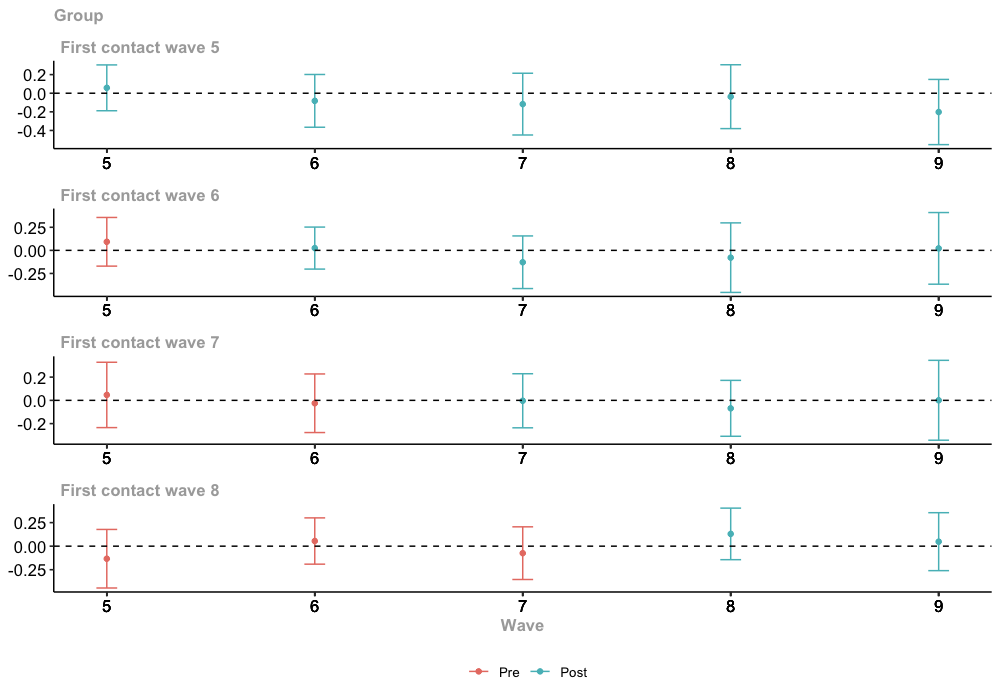


**Figure G4**. Group-time ATT for females


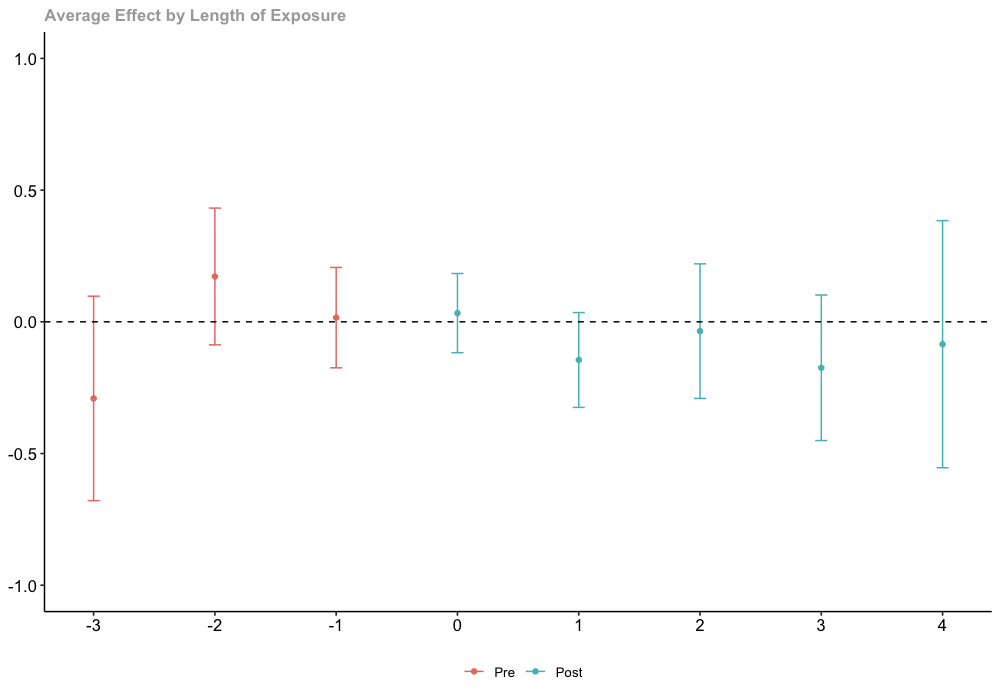


**Figure G5**. Results aggregated by dynamic/event-study ATT for females

**
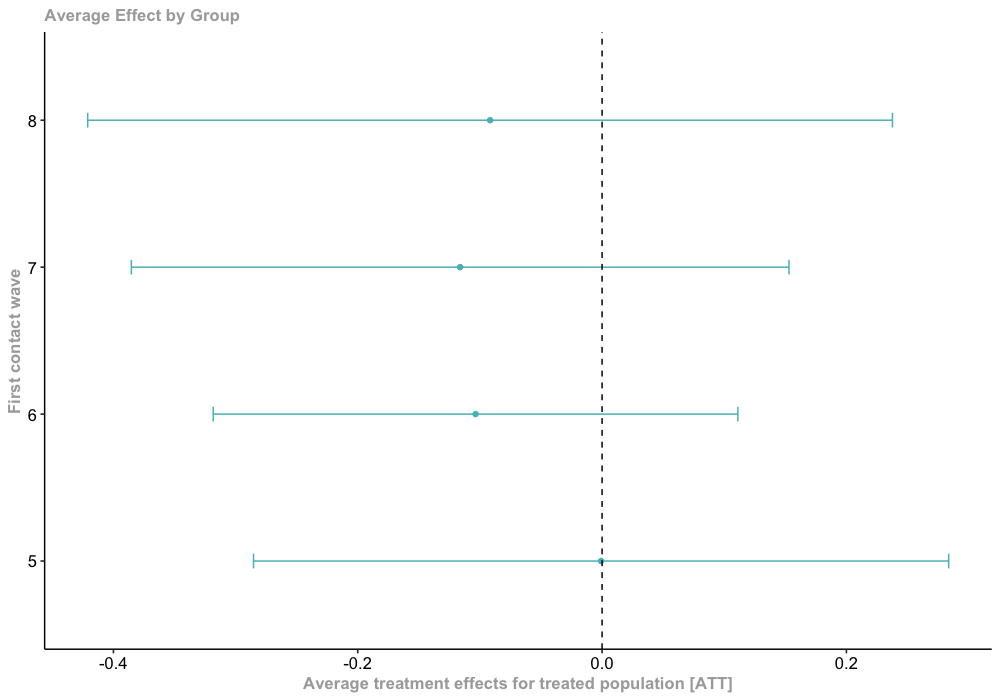
**

**Figure G6**. Results aggregated by group ATT for females

# Appendix H – Disaggregated results by migrant background

All analyses were conducted using the same process as the main analyses, excluding covariates. The pre-test for parallel trends was not significant for both groups (p=0.241 for non-migrant background, p=0.476 for migrant background).


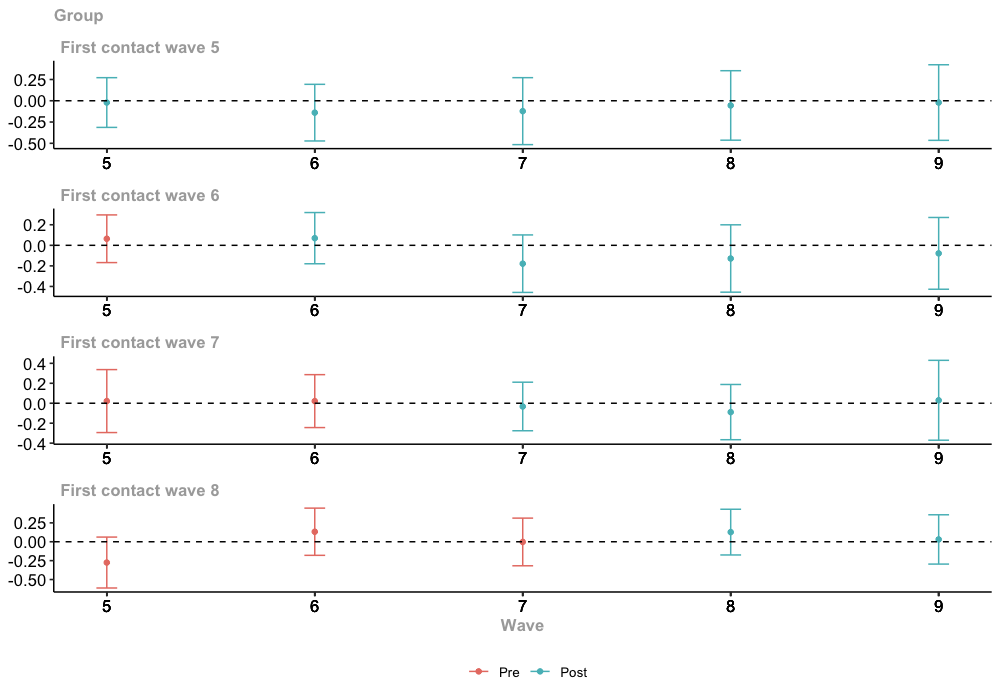


**Figure H1**. Group-time ATT for participants without a migrant background


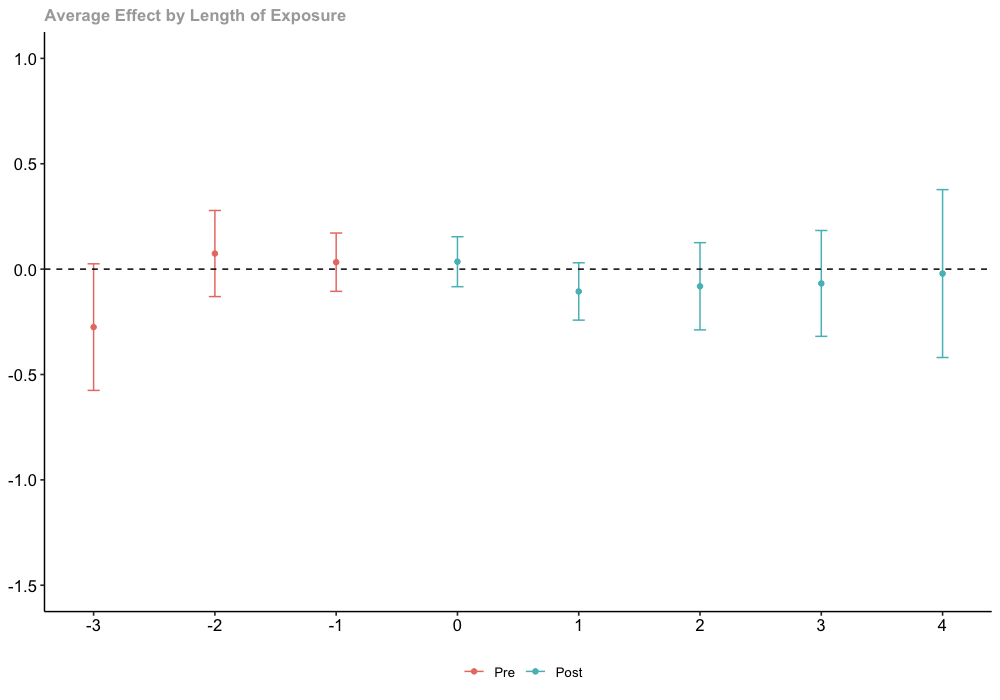


**Figure H2**. Results aggregated by dynamic/event-study ATT for participants without a migrant background


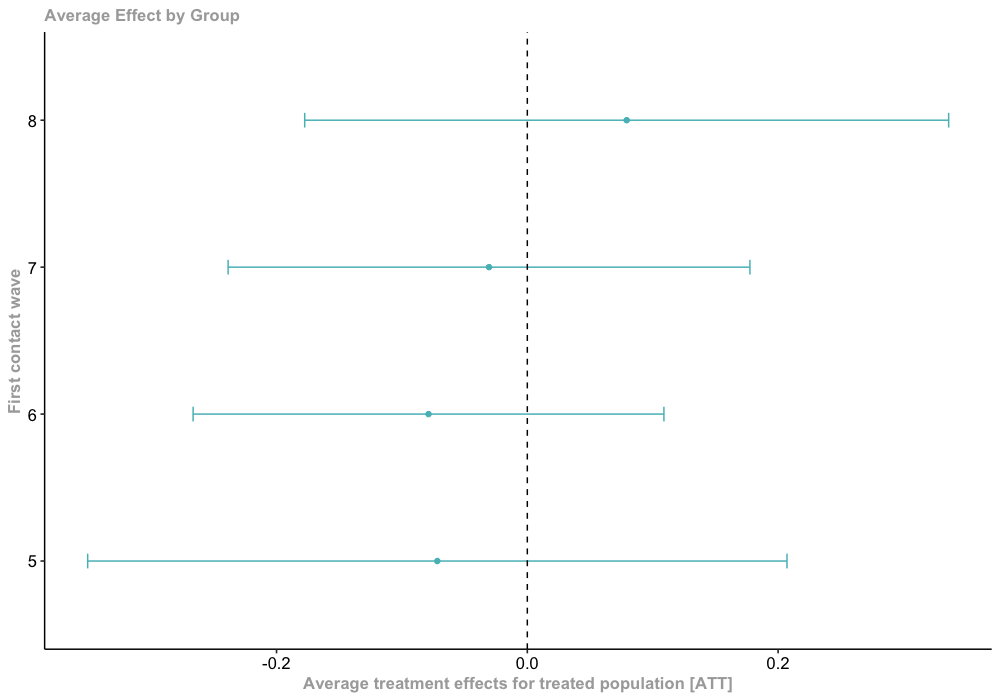


**Figure H3**. Results aggregated by group ATT for participants without a migrant background


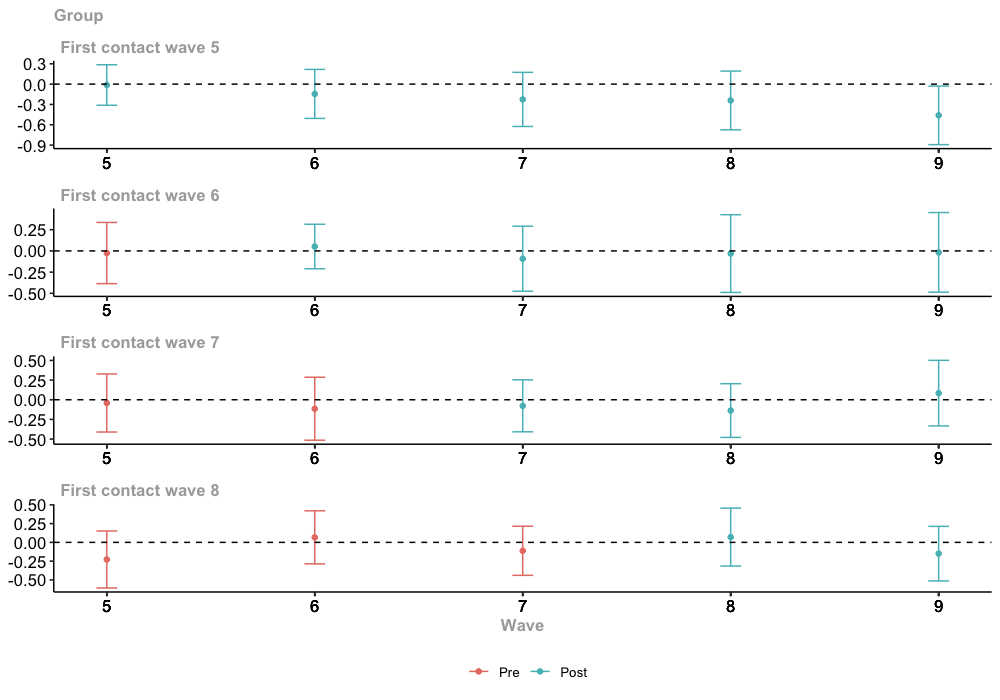


**Figure H4**. Group-time ATT for participants with a migrant background


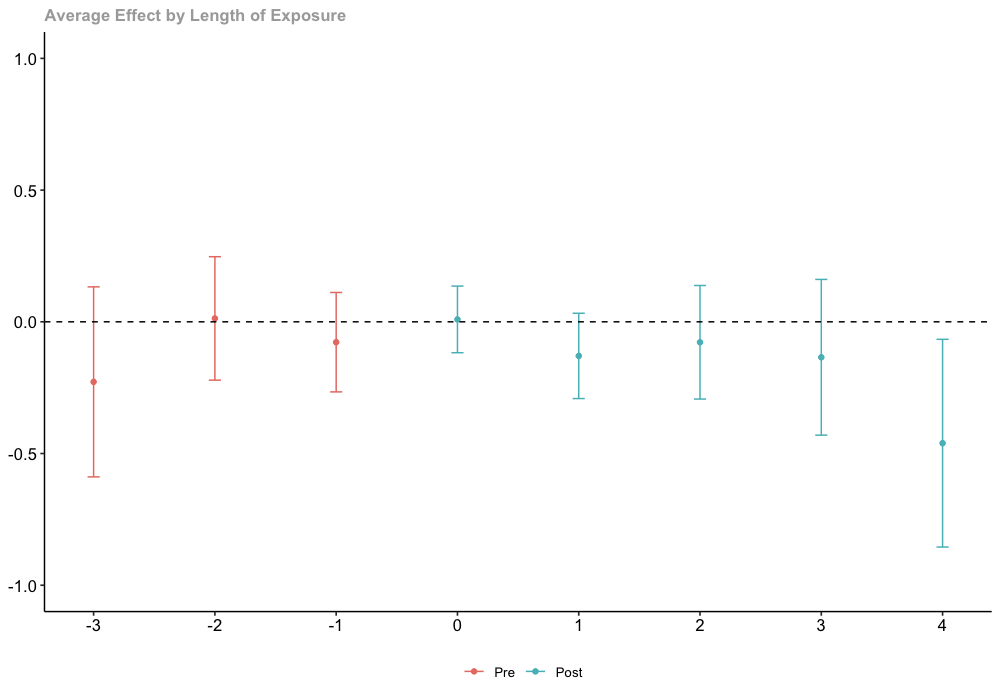


**Figure H5**. Results aggregated by dynamic/event-study ATT for participants with a migrant background


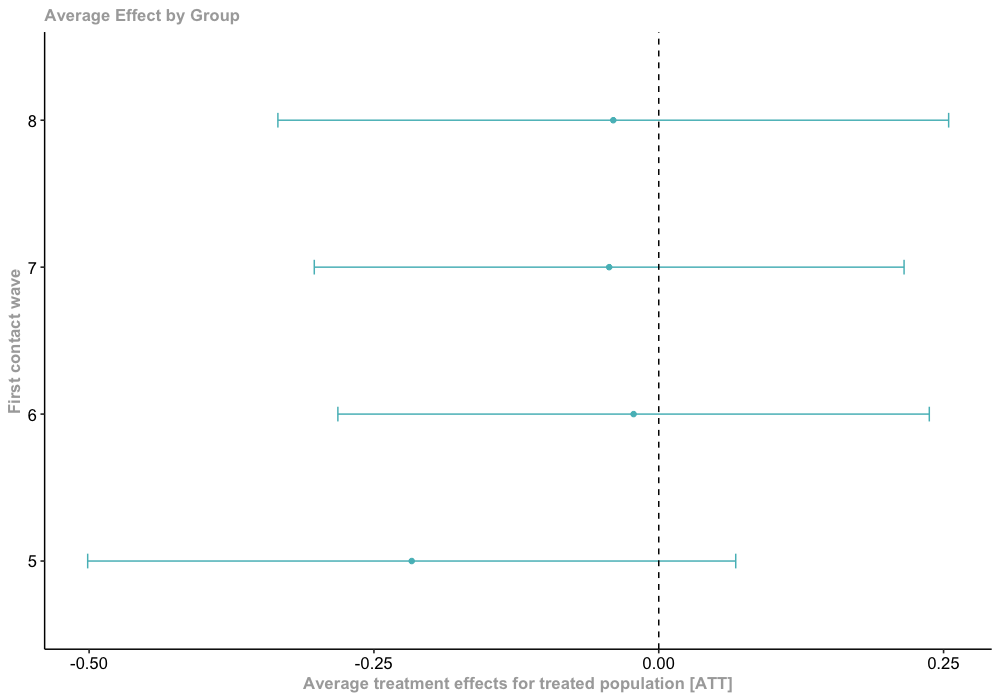


**Figure H6**. Results aggregated by group ATT for participants with a migrant background

# References

Austin, P. C. (2011). An Introduction to Propensity Score Methods for Reducing the Effects of Confounding in Observational Studies. *Multivariate Behavioral Research*, *46*(3), 399–424. https://doi.org/10.1080/00273171.2011.568786

Copeland, W. E., Shanahan, L., Costello, E. J., & Angold, A. (2009). Childhood and Adolescent Psychiatric Disorders as Predictors of Young Adult Disorders. *Archives of General Psychiatry*, *66*(7), 764. https://doi.org/10.1001/archgenpsychiatry.2009.85

Croissant, Y., & Millo, G. (2008). Panel Data Econometrics in R: The plm Package. *Journal of Statistical Software*, *27*(2), 1–43. https://doi.org/10.18637/jss.v027.i02

Dennison, C. R., & Finkeldey, J. G. (2021). Self‐reported experiences and consequences of unfair treatment by police ^*^. *Criminology*, *59*(2), 254–290. https://doi.org/10.1111/1745-9125.12269

Ho, D. E., Imai, K., King, G., & Stuart, E. A. (2011). MatchIt: Nonparametric Preprocessing for Parametric Causal Inference. *Journal of Statistical Software*, *42*(8), 1–28.

Jackson, D. B., Testa, A., Fix, R. L., & Mendelson, T. (2021). Adolescent Police Stops, Self-Harm, and Attempted Suicide: Findings From the UK Millennium Cohort Study, 2012‒2019. *American Journal of Public Health*, *111*(10), 1885–1893. https://doi.org/10.2105/AJPH.2021.306434

Jackson, D. B., Testa, A., & Vaughn, M. G. (2020). Low self-control and the adolescent police stop: Intrusiveness, emotional response, and psychological well-being. *Journal of Criminal Justice*, *66*, 101635. https://doi.org/10.1016/j.jcrimjus.2019.101635

Murray, A. L., Eisner, M., & Ribeaud, D. (2020). Within‐person analysis of developmental cascades between externalising and internalising problems. *Journal of Child Psychology and Psychiatry*, *61*(6), 681–688. https://doi.org/10.1111/jcpp.13150

Nguyen, T.-L., Collins, G. S., Spence, J., Daurès, J.-P., Devereaux, P. J., Landais, P., & Le Manach, Y. (2017). Double-adjustment in propensity score matching analysis: Choosing a threshold for considering residual imbalance. *BMC Medical Research Methodology*, *17*(1), 78. https://doi.org/10.1186/s12874-017-0338-0

Wiley, S. A., Slocum, L. A., & Esbensen, F.-A. (2013). THE UNINTENDED CONSEQUENCES OF BEING STOPPED OR ARRESTED: AN EXPLORATION OF THE LABELING MECHANISMS THROUGH WHICH POLICE CONTACT LEADS TO SUBSEQUENT DELINQUENCY: LABELING MECHANISMS AND SUBSEQUENT DELINQUENCY. *Criminology*, *51*(4), 927–966. https://doi.org/10.1111/1745-9125.12024
